# A Trust-Based Secure Routing Scheme Using the Traceback Approach for Energy-Harvesting Wireless Sensor Networks

**DOI:** 10.3390/s18030751

**Published:** 2018-03-01

**Authors:** Jiawei Tang, Anfeng Liu, Jian Zhang, Neal N. Xiong, Zhiwen Zeng, Tian Wang

**Affiliations:** 1 School of Information Science and Engineering, Central South University, Changsha 410083, China; tangjiawei@csu.edu.cn (J.T.); afengliu@mail.csu.edu.cn (A.L.); zengzhiwen@mail.csu.edu.cn (Z.Z.); 2 Department of Mathematics and Computer Science, Northeastern State University, Tahlequah, OK 74464, USA; xiongnaixue@gmail.com; 3 College of Computer Science & Technology, Huaqiao University, Xiamen 361021, China; wangtian@hqu.edu.cn

**Keywords:** wireless energy harvesting networks, security, disjoint routing, marking, network lifetime

## Abstract

The Internet of things (IoT) is composed of billions of sensing devices that are subject to threats stemming from increasing reliance on communications technologies. A Trust-Based Secure Routing (TBSR) scheme using the traceback approach is proposed to improve the security of data routing and maximize the use of available energy in Energy-Harvesting Wireless Sensor Networks (EHWSNs). The main contributions of a TBSR are (a) the source nodes send data and notification to sinks through disjoint paths, separately; in such a mechanism, the data and notification can be verified independently to ensure their security. (b) Furthermore, the data and notification adopt a dynamic probability of marking and logging approach during the routing. Therefore, when attacked, the network will adopt the traceback approach to locate and clear malicious nodes to ensure security. The probability of marking is determined based on the level of battery remaining; when nodes harvest more energy, the probability of marking is higher, which can improve network security. Because if the probability of marking is higher, the number of marked nodes on the data packet routing path will be more, and the sink will be more likely to trace back the data packet routing path and find malicious nodes according to this notification. When data packets are routed again, they tend to bypass these malicious nodes, which make the success rate of routing higher and lead to improved network security. When the battery level is low, the probability of marking will be decreased, which is able to save energy. For logging, when the battery level is high, the network adopts a larger probability of marking and smaller probability of logging to transmit notification to the sink, which can reserve enough storage space to meet the storage demand for the period of the battery on low level; when the battery level is low, increasing the probability of logging can reduce energy consumption. After the level of battery remaining is high enough, nodes then send the notification which was logged before to the sink. Compared with past solutions, our results indicate that the performance of the TBSR scheme has been improved comprehensively; it can effectively increase the quantity of notification received by the sink by 20%, increase energy efficiency by 11%, reduce the maximum storage capacity needed by nodes by 33.3% and improve the success rate of routing by approximately 16.30%.

## 1. Introduction

Ubiquitous sensor-based devices (e.g., sensor nodes, wearable sensing devices, and smartphones) [1,2,3,4,5] have been playing a vital role in the evolution of the Internet of Things (IoT) [2,4,5,6,7,8,9], which bridges the gap between digital and physical spaces [6,7,8]. However, the energy issue of sensor terminals poses significant challenges to the widespread use of IoT, in which the sensor devices generally have small volume and battery with limited capacity [10,11,12,13,14,15]. Therefore, the sustainable issue of IoT has attracted considerable attention from both academia and industry [16,17,18,19]. Wireless energy harvesting and transfer technology was recently proposed as an effective mean to address this issue. Energy-Harvesting Wireless Sensor Networks (EHWSNs) refer to networks whose nodes can collect and complement energy by relying on the ambient environment (such as solar energy, wind energy, thermal energy and vibration energy) [20,21]. EHWSNs are able to charge themselves via renewable resources; thus, they can be applied to unattended but important and complex environments for long-term (even permanent) monitoring. These networks are called green networks because they use renewable energy and cause less interference or damage to the ambient environment [21,22,23]. For the above reasons, EHWSNs have widely gained the attention of researchers and are especially suitable for applications in the national economy, national defense and military, battlefield protection, protection of wide and rare animals and medical and health monitoring [24,25,26]. Security has been always a critical point in the development and application of sensor networks [6,10,27,28,29,30,31]. For EHWSNs, there are three issues that be taken into consideration in designing the secure routing scheme:

(1) The core of IoT lies in collecting data and enabling data communication between the required nodes to form a coordinated communication network. Therefore, a blocking communication attack that blocks the communication between nodes is a harmful and effective attack behavior [30,31,32]. Existing research shows that over 30 types of blocking communication attack behaviors or strategies have been found for wireless sensor networks. These attack behaviors primarily include black attack [30,31], clone attack [32], Dos attack [30,31], selective forwarding attack [33,34,35] and false data injection attack [34]. These attacks can not only block network communication but also consume the energy of limited sensor nodes, causing the earlier death of the network [36].
(2) Although many routing schemes can resist the security attacks, most defenses are conducted against one type of attack behavior. In other words, a specific scheme only works for one specific type of attack but does not work or works with limited effect for other types [27]. The attack methods and technologies are constantly advancing, so the resistance method against a specific type of attack behavior usually performs less satisfactorily in practice.
(3) The secure routing scheme tends to consider other performances of the network. For example, energy consumption is an important performance metric for sensor-based IoT [37]. Due to the limited battery capacity of sensor-based devices, how to minimize the energy consumption of a network is an important issue in the context of ensuring network security [38,39,40,41,42]. Although the pressure for reducing energy consumption is relieved in the case of energy-harvesting wireless networks, how to reduce energy consumption remains an important issue to be researched because, although energy-harvesting networks can harvest energy from environment, doing so requires extra energy collection hardware. Networks are expected to minimize the cost of the energy collection hardware because of the requirement to reduce the manufacturing cost [20,23,25]. Therefore, overall, even sensor devices with energy harvesting cannot obtain unlimited energy compensation, and the effective utilization of energy remains a severe challenge. Thus, improving network performance is necessary [43,44]. In addition, an energy-harvesting network has another important feature, i.e., when sufficient external energy compensation can be provided, the complemented energy will be fully utilized to improve the network performance, but it is not a good scheme to merely save energy. Thus, in EHWSNs, the power management was usually modeled as energy neutral operation [23,24], which maximizes the utilization of the energy absorbed from the ambient environment and achieves the balance between the energy consumption of the system and the absorbed energy. The features that determine the design and schemes of their secure routing are obviously different from the conventional schemes, which also bring great challenges. For this reason, how to achieve efficient and safe routing in EHWSNs is rarely researched. After a deep analysis on EHWSNs, a trust-based secure routing (TBSR) scheme using the traceback approach is proposed to improve the security of data routing and maximize the use of available energy in energy-harvesting wireless sensor networks (EHWSN). The main contributions of this paper are as follows:
  (1) A data and notification disjoint routing approach is proposed for improving the security of networks. In this approach, the source node sends data and notification to the sink through disjoint paths separately; in such a mechanism, the data and notification can be verified independently to ensure their security.
  (2) A traceback approach is integrated into the TBSR scheme, which can trace malicious nodes more effectively than ordinary wireless sensor networks. In the TBSR scheme, the data and notification adopt a probability-based marking and logging approach during the routing. Therefore, when attacked, the network will adopt the traceback approach to locate and clear malicious nodes to ensure security. In a traceback scheme, the higher the probability of marking is, the safer the system will be, but more energy will be consumed and the network lifetime will be affected. In the TBSR scheme, the probability of marking is determined based on the level of battery remaining. When the level of battery remaining is high, the probability of marking is higher, which can improve the network security. When the battery level is low, the probability of marking will be decreased, which is able to save energy. For logging, when the battery level is high, the network adopts a larger probability of marking and smaller probability of logging to transmit notification to the sink, which can reserve enough storage space to meet the storage demand for the period of the battery on low level; when the battery level is low, increasing the probability of logging can reduce energy consumption. After the level of battery remaining is high enough, nodes then send the notification which was logged before to the sink. In this paper, we discuss the two cases “the battery on low level” and “the battery on high level” separately, which can enhance the overall network security. If we not, the probability of marking and logging will not be changed. However, in order to maintain the level of battery remaining above 0 or a lower limit at any time, the network will adopt the probability of marking and logging in accordance with the case of “the battery on low level,” so the probability of marking is lower. The sink will receive less notification and find malicious nodes slower, so the network security will be lower.
  (3) Compared with past schemes, our results indicate that the performance of the TBSR scheme has been improved comprehensively; it can effectively increase the quantity of notification received by the sink by 20%, increase energy efficiency by 11%, reduce the maximum storage capacity needed by nodes by 33.3% and improve the routing success rate by approximately 16.30%.

The rest of this paper is organized as follows: in Section 2, related works are reviewed. The system model is described in Section 3. In Section 4, a novel TBSR scheme is presented. Performance analyses and experimental results of TBSR are provided in Section 5. We conclude in Section 6.

## 2. Related Work

Much research has been conducted on the secure routing schemes of wireless sensor networks. This section is divided into the following 3 parts to introduce the works related to this paper: (1) schemes and approaches related to secure routing [30,31,32,33,34,35,45,46]; (2) routing schemes related to traceback [47,48,49]; (3) energy consumption features of Energy-Harvesting Wireless Sensor Networks (EHWSNs) and management-related schemes [20,21,22,23,24,25,26].

(1) Strategies and approaches related to secure routing. Secure routing means adopting proper strategies or approaches to successfully transmit the data produced by source nodes to the sink or an ability to resist a security attack [30,31,32,33,34,35]. Its purpose is to ensure the successful transmission of data to the sink with a high probability even in the event of an attack. This paper classifies secure routing mechanisms into the following types:
  (a) The first type of secure routing scheme cannot detect whether an attacker exists in the network or whether the transmission is attacked. These schemes largely adopt the strategy of multiple redundant routings, i.e., one data packet is transmitted to the sink through 2 or more routing paths. In this case, even when an attack behavior exists in the network, the probability of the multiple routing paths being attacked simultaneously is much lower than that of only one routing path being attacked. Thus, the probability of successfully sending the data to the sink can be improved effectively. The advantages of these schemes are that they have wide applicability and can be used in all types of applications, have fewer network requirements and present favorable effects in resisting various attack behaviors. However, the disadvantages are that each data packet is sent through multiple redundant routing paths; thus, energy consumption will be high, which affects network lifetime. Moreover, no detection mechanism is adopted to determine whether the routing is attacked, so the strategy is inflexible and cannot bypass the routing path even after it has been attacked. For relevant research, please see the multi-path routing approach proposed by Karlof et al. [50] and the SEDR scheme proposed by Reference [31].
  (b) The second type of routing schemes introduces the following improvements based on the first type: multiple routing paths will consume additional energy and therefore significantly affect network lifetime. Thus, sequential routing schemes try one routing path first and, if the routing fails, transmit the data through another, different routing path, which improves the probability of the data successfully reaching the sink. For example, the multi-dataflow topology (MDT) scheme proposed by Hung-Min Sun et al. [51] is representative of this type of scheme. In the MDT scheme, the network is divided into two disjoint topology structures, and a node can send the data to the sink through any topology structure. Therefore, if the source node fails to send the data through one topology structure, it can resend the data through the other topology structure unless the attacker simultaneously attacks 2 topology structures, which will cause routing failure. Obviously, there is a much lower probability of the attackers simultaneously attacking two topology structures, so the MDT scheme can effectively improve routing security. Compared with past schemes, such routing schemes have the advantage of low cost, i.e., they do not require sending data simultaneously through m routing paths, which saves energy and lifts efficiency. The schemes’ disadvantages include that they cannot identify and locate malicious nodes or adapt themselves to improve the success rate of routing and are weak in resisting intelligent attackers. Their cost and energy consumption are also significant. For example, the MDT scheme requires constructing multiple topology networks simultaneously, which increases the requirements for the network and the costs of construction.
  (c) The purpose of the third type of routing scheme is adopting a proper mechanism to detect whether the routing is successful and identifying and locating the position of malicious nodes, thereby increasing the success rate of routing as time passes. For example, a checkpoint-based multi-hop acknowledgement (CHEMAS) scheme is proposed by Xiao, B et al. [33] for identifying suspect nodes. In the CHEMAS scheme, some nodes on the routing path from the source node to the sink are selected as check nodes. When each check node receives the data, it will return the ACK information in the data-source direction. If the data packets are attacked, the check node will fail to receive the pre-defined number of ACKs and recognize that malicious nodes exist on the routing path. Finally, the position of malicious nodes can be largely determined by the different number of ACKs received by different check nodes. Obviously, the scheme has suppressive effects on malicious nodes and can guide data transmission to avoid the position of malicious nodes during the next routing. The administrators can even remove the malicious nodes physically through powerful strategies. However, the CHEMAS scheme also has disadvantages. In the CHEMAS scheme, the ACK information is returned along the original routing path of data instead of via an independent path, so it will also be attached by the attacker. Another commonly used scheme is a trust-based strategy. ActiveTrust [30] is a good secure routing scheme proposed for wireless sensor networks and is based on active trust. In the ActiveTrust scheme, the remaining energy in the remote sink is fully utilized to initiate a detective routing. A detective routing is not a real data routing, but it is the same as the real routing. Therefore, malicious nodes will attack the detective routing as it does a data routing; thus, the suspected hostile nodes will be exposed. The trust for suspected and normal nodes will be lowered and lifted respectively. As this process proceeds, the trust for malicious nodes will become lower and that for normal nodes will become higher to allow the routing to effectively improve the success rate of routing by selecting nodes with high trust. The scheme performs well in defending intelligent malicious nodes and resisting various attacks and has high energy efficiency and recognized significance.
(2) Relevant research on Traceback. The Traceback approach is also an effective approach to improve network security [27,47,48,49]. The important difference between Traceback and the conventional approaches is that it saves the path information of nodes during the routing process so that it can reconstruct the path of the attacker when the network is attacked to identify the malicious nodes, then notify the system and remove these malicious nodes physically, ensuring network security. Multiple traceback approaches have been proposed, and most are based on the following 2 traceback schemes: (a) Marking-based traceback scheme (also known as marking scheme) [47], and (b) Logging-based traceback scheme (also known as logging scheme) [48].
  (a) Marking-based traceback scheme. Actually, marking is the main strategy of traceback [47]. It adopts a method in which all nodes on the routing path attach their node ID and other information to the data packet during the routing process (the information attached to the data packet is called notification). When the network is attacked, the path from the source node to the sink can be reconstructed by extracting the notification. Combining the data from multiple source nodes can determine the scope of malicious nodes with a very high probability and achieve the purpose of tracing the malicious nodes.

The advantage of a marking-based traceback scheme is that it has lower network requirements and can be used for both wired and wireless networks. However, its most apparent disadvantage is that the energy consumption of the wireless sensor network is affected significantly; therefore, the network lifetime is shortened because in the marking scheme, a basic marking unit will be added to the transmitted marking once the routing data passes through a node. As the routing continues, the length of the data packet becomes long, and an increasing amount of data will be transmitted by sensor nodes. The nodes in the area near the sink carry much more data than those far from the sink. After the marking scheme is adopted, the local notes will load the data multiple times compared with the data loaded by nodes in areas far from the sink. In this case, the unbalance of network energy consumption is aggravated and the lifetime is significantly shortened. To reduce the damage caused by the notification to network energy, some researchers propose a probability-based marking scheme, which changes the scheme of marking every node in the conventional schemes and adopts a scheme of marking each node based on probability. The probability-based marking scheme has an advantage of effectively reducing the number of marking nodes and the energy consumption of the system to transmit the notification [47]. Conversely, the scheme has a lower ability to trace malicious nodes. In this scheme, the marking is not added for every node, so some nodes will be omitted during the reconstruction of a routing path from the source node to the sink; therefore, the routing data for such omitted nodes must be contained in other collected data to construct the complete path. However, collecting more data requires a long time, i.e., prolongs the convergence time, which is one of the important indexes of scheme performance. Reference [52] proposed an improved scheme against such a case. The main idea of their scheme is adopting different marking probabilities based on the security status of the network. When the network is secure, a smaller probability of marking is adopted; when the number of network attacks is increasing, the probability of marking is also increased correspondingly. The network is usually secure, so a smaller probability of marking is usually adopted and the greater probability of marking is only adopted for short periods. Therefore, the overall effect is that network security can be effectively guaranteed, the number of marking nodes is not large, and network lifetime is long.

(b) Logging-based traceback scheme. The logging-based scheme is another malicious node tracing technology [48]. The above introduction shows that the marking-based traceback approach adds many loads to the network, which affects the network lifetime. This logging scheme adopts the following approach to reduce the effect of notification on the network lifetime. Its essential idea is that each node in the network has a fixed storage capacity. Therefore, the storage capacity of nodes in the network can be fully utilized to store the notification on these nodes instead of sending it to the sink. When the network is attacked, these nodes will be requested to send the notification to the sink for traceback. Then, the traceback path can be reconstructed. Therefore, the specific approach to adopt the logging scheme is that the node adds the notification to the passing data packet with a certain probability, and when the quantity of notification in the data packet reaches a certain value, such as k, all notification will be recorded on nodes through the logging process. The notification that has been recorded on nodes will not be forwarded during the routing of subsequent data packets to the sink. The adopted scheme can effectively reduce the amount of data to be transmitted by the network and save network energy. CPMLT (combined packet marking and logging scheme for traceback) [53] is a representative of this type of scheme.

Although logging scheme can reduce the energy consumption of a network, the reduction is achieved at the cost of node storage space. Therefore, this type of scheme requires a certain storage capacity. In addition, the unbalanced utilization of storage capacity remains in the wireless sensor network, i.e., the storage space of nodes far from the sink area is not fully utilized, but that of nodes near the sink area is insufficient. This shortfall exists because the nodes constantly store the notification during the routing to the sink; thus, more notification should be recorded by logging near the sink area, and less should be recorded far from the sink area.

In the traceback approach, the key to reconstructing the traceback path is to obtain more notification better. Therefore, both marking and logging schemes are trying to obtain as much notification as possible. Reference [54] analyzed and obtained the general traceback approach in which a serious unbalance exists in the network between the consumption of energy and storage consumption. Specifically, the unbalance is that more energy and storage space are consumed in nodes near the sink area, but nodes far from the sink area have much remaining battery level and storage space. In view of this case, Reference [54] proposed a logging and migrating (LM) traceback scheme because the non-hotspot areas in the sensor network have over 90% remaining battery level and storage space, but the remaining storage space and battery level are insufficient near the sink area. In the LM traceback scheme, the marking data packets log all their notification on the nodes before approaching the hotspot area, and the non-hotspot nodes have remaining battery level and storage space, so logging the notification in the non-hotspot areas in advance will greatly reduce the pressure of battery level and storage space in the hotspot area. Moreover, the nodes near the hotspot areas store much notification, so when the storage space is insufficient, the notification logged in these areas will be migrated to remoter nodes with remaining space, which significantly improves the amount of notification stored by the system compared with the conventional schemes. Thus, the scheme performs well in lengthening network lifetime and storing notification.

(3) Energy Consumption Features of Energy-Harvesting Wireless Sensor Networks (EHWSNs) and relevant Management Schemes

Energy has always been the key research issue for wireless sensor networks because of their limited battery capacity and lifetime of the ordinary wireless sensor network. EHWSN is an effective solution proposed to address the limited battery capacity of the sensor network. In EHWSNs, in addition to the components for ordinary sensor nodes, hardware equipment able to absorb energy from the ambient environment is added to the sensor. The most important sensor network energy absorbed from the ambient environment is solar power in wireless sensor networks in which sensor nodes are provisioned with a solar panel and battery combination [21]. The solar panel is usually photovoltaic (PV), and the battery is rechargeable. The panel can absorb energy from the ambient environment; thus, the energy management scheme of EHWSNs is significantly different from the traditional WSNs. In WSNs, the main goal is to reduce the energy consumption, whereas in EPWSNs, the main purpose is to efficiently utilize available energy instead of reducing energy consumption [21]. This purpose exists because in EHWSNs such as solar-powered WSNs, when the solar radiation is strong, the nodes can absorb a large amount of energy but cannot store all of the energy due to the limited battery storage capacity of nodes. In this case, the nodes need not save energy and should make as full use of the energy as is possible. They use the energy for various operations, such as receiving and transmitting energy and system maintenance, and store sufficient energy for use at night without solar energy. Therefore, much research [23,24] notes that the new guiding principle in EHWSNs is energy-neutral operation, which consists of two simultaneous goals: (i) optimizing the network performance but (ii) ensuring that energy supply and energy demand are balanced [21,23,25].

Much research has been conducted on the energy management of EHWSNs. This research includes multiple aspects. First, in terms of hardware, for solar-powered wireless sensor networks, the size of the solar panel is an important issue. When the solar panel is too large, sufficient energy can be supplied, but more manufacturing costs will be required. When the solar panel is too small, insufficient energy is supplied to nodes. Therefore, Reference [21] propose an energy management algorithm based on shortest-path routing to minimize the network deployment cost (primarily the size of the solar panel) for a given energy source assignment.

When EHWSNs adopt the given hardware configuration, more research explored how to make full use of energy without an outage when optimizing network performance. Network performance indexes primarily include for example delay and channel throughput. The optimizations of these performance indexes are all closely related to energy. Thus, many studies have been conducted on this topic.

Duty cycle is an energy-saving mechanism adopted by and widely applied in most sensor networks. In this mechanism, the node sleeps and awakes periodically; when the node is in sleep status, its energy consumption is only 1/1000 that in awake status. Therefore, nodes will remain in sleep status as much as possible to save energy. However, a long sleep time aggravates network performance. The main effect lies in aggravating network delay and the network’s ability to monitor the environment. The sensor node cannot send or transmit data in sleep status or monitor the ambient environment. It cannot send or transmit data, so the routing from source to sink requires a long delay. It cannot monitor the ambient environment, so important events and objectives might be missed during the monitoring. Obviously, the duty cycle also has an important effect on channel throughput. When the duty cycle is long, nodes can send and receive data for a longer time and process a greater amount of data, which will improve the channel throughput of the network. Therefore, some researchers proposed effective studies to optimize the performance of EHWSNs. The main ideas of this research are as follows: (a) propose an effective energy prediction model and scheme to make full use of the available energy; (b) dynamically change the duty cycle of nodes, i.e., maximize the duty cycle when battery level on high level to optimize network performance and select the optimal duty cycle when the battery on low level.

First, modeling the energy absorbed by nodes is the basis for the success of these schemes. The general principle is that if the energy that the nodes can absorb in a coming period can be predicted, the energy use can be planned in advance and the energy utilization can be maximized to optimize network performance. For example, there is more solar energy radiation in the sunny daytime. If it is predicted that more energy will be absorbed in the future, the remaining battery level can be thoroughly consumed in advance because sufficient energy compensation will be provided subsequently. If it is predicted that less energy can be absorbed in the coming period, some energy should be stored for the future (night) to meet the energy consumption requirements before the next replenishment opportunity. Peng et al. [23] used a finite state Markov model and general stochastic model to model the energy-harvesting process in Reference [24].

Second, the maximum energy consumption of the current node can be calculated based on the prediction for energy. With a calculated result, the duty cycle (or sleep and wake) can be dynamically adjusted according to the available energy to optimize network performance [25]. When the energy is sufficient, the duty cycle of nodes will be maximized so that the delay and channel throughput of the network can be effectively optimized [26].

The above discussions show that opportunities remain for further research on the secure routing of EHWSNs. First, the secure routing is greatly different for WSN networks and EHWSNs. The secure routing for WSNs has limited functions and weak resistance against attacks due to the limited battery capacity. Moreover, secure routing schemes do not consider the full utilization of energy in EHWSNs. Therefore, effective secure routing for EHWSNs is obviously more important. Second, past secure routing schemes usually contain only one secure scheme. To the best of our knowledge, there is no scheme combining the secure routing scheme and the traceback scheme. Past traceback schemes were primarily proposed for WSNs, but this paper proposes a new secure routing in combination with the traceback scheme to further improve network security and other aspects of network performance. Finally, the traceback scheme is very suitable for EHWSNs, which can make full use of the storage space and the absorbed energy to improve the effectiveness of the traceback scheme. Based on the above analysis, this paper proposes a new secure routing scheme that is highly effective for EHWSNs.

## 3. System Model and Problem Statement

### 3.1. System Model

***A network model***

The network model in this paper is a typical planar periodic data collection wireless sensor network similar to [27,55,56,57]. Its system model is as follows:

(1) There are n homogeneous sensor nodes which are randomly deployed in a two-dimensional planar network with a radius of R, a sink is at the center and the node density is ρ. The node communication radius is r [2,56].
(2) The size of a data packet and notification are set to m bytes and b bytes respectively. The success rate of each hop is set to p, the initial battery level and the maximum battery level of each sensor node was set to $E_{initial}$ and $E_{max}$ respectively.

***B Energy-harvesting node model***

**Figure 1:**
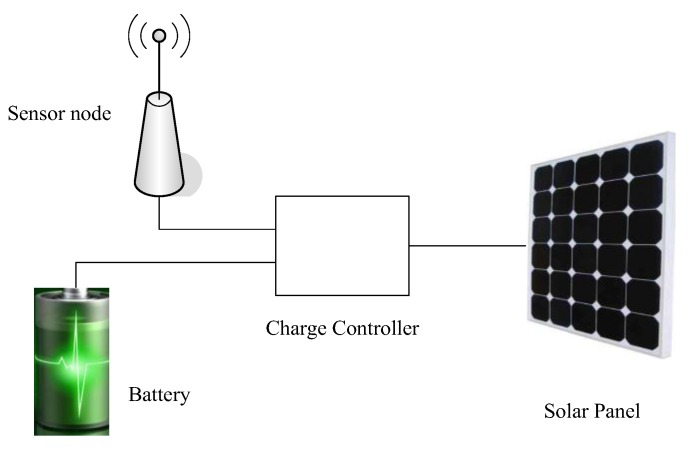
Solar node structure diagram.

In EHWSNs, sensor nodes are usually divided into five parts: a processor module, a sensor module, a wireless communication module, a solar collector, and a battery, the power controller as shown in Figure 1. Its processor module, sensor module and wireless communication module are the same as the modules in an ordinary sensor network [2,5]. Its solar collector, battery module and power controller are different from those in traditional sensor nodes. The solar collector module is an energy-harvesting node model; its function is converting solar energy to electrical energy through the photovoltaic or chemical effect. The battery is the power supply module of the system. It stores the electrical energy collected by solar collector and has a limited capacity. When the battery is not fully charged, the solar collector can charge it. When the battery level is full, the solar collector cannot charge it even when it collects more electrical energy. The power controller is the control system for electrical energy. It adjusts the transmission frequency of the wireless communication module based on the level of battery remaining, sun exposure time, intensity of sunlight, and day and night relationship to change the energy consumption of the wireless sensor and maximize the utilization of limited electrical energy.

### 3.2. Data Aggregation Model

This paper adopts a typical data aggregation model similar to that described in the literature [58]. In such a data aggregation model, when the network collects data, some nodes are selected as aggregators and other nodes are simple nodes. Each simple node determines which aggregator it belongs to with the clustering algorithm in Reference [58] and then sends its own data packet to the aggregator directly. If the simple node $S_{i}$ belongs to aggregator $S_{j}$, the simple node is called a member node of the aggregator. The aggregator $S_{j}$ aggregates data packets sent by all member nodes into one data packet.

When aggregator $S_{j}$ receives the data packet sent by a member node $S_{i}$, it will aggregate the data packet $D_{i}$ sent by $S_{i}$ and the existing data packet $S_{j}$ of aggregator $S_{j}$ ($S_{j}$ might be the original data packet $D_{j}$ of aggregator $S_{j}$ or an intermediate result $S_{j}$ during the data aggregation of member nodes by aggregator $S_{j}$, collectively expressed with $S_{j}$). $X\left(S_{i},\;S_{j}\right)$ is used to indicate the final result of the data aggregation of two nodes $S_{i},\;S_{j}$. The calculation formula is as follows:

(1) $$X\left(S_{i},\;S_{j}\right)=\max\left(D_{i},\;S_{j}\right)+\left(1-c_{i,\;j}\right)\min\left(D_{i},\;S_{j}\right)$$

where $c_{i,j}$ is the correlation coefficient between nodes $S_{i}$ and $S_{j}$. A larger $c_{i,j}$ indicates a higher correlation between the data of nodes and a smaller length of data packet formed after the data aggregation.

### 3.3. Security Model

This paper assumes that the attacker tends to be very intelligent. The security attack against the network is largely blocking and dropping data packets in the network, thus damaging the functions of the network. For example, the sink cannot react to the monitored events in the network if it fails to receive the monitored data packets, so the harmful event will cause serious loss to the network. Blocking the routing of some important data packets will cause an incorrect decision of the sink because it fails to receive sufficient notification. For example, the attacker adopts a proper operational mode to capture a small part of data, steals and modifies the program in the part, which helps the attacker control the nodes that have obtained legal status and allows it to lodge various attacks. The Attacker is able to drop data packets with a certain probability (if the drop probability is 1, then it is a black hole attacker; otherwise, it is likely to be a selective forwarding attacker or a Denial of Service (DOS) attacker) and cause maximum harm to the network without exposing its own identity. On the one hand, attackers can also forge real nodes to launch various attacks, such as false data injection attacks. On the other hand, attackers can also collude to launch attacks, making the problem more complicated. However, if most nodes in a network are malicious nodes, network safety cannot be guaranteed [58]. Therefore, in this paper, we assume that the proportion of malicious nodes is small, for example, less than ς.

### 3.4. Energy Consumption Model

In this paper, we adopt the simplified X-MAC energy consumption model. X-MAC belongs to asynchronous competition MAC protocols. In these protocols, all nodes maintain their own duty cycle, and the transmitter and receiver are asynchronous. Thus, the receiving node might be in sleep status when the sending node sends the data out, and the LPL (Low-Power Listening) leader sequence technology will be adopted to wake up the receiving node. Therefore, in the X-MAC energy consumption model, the energy consumption power of each sensor node primarily includes the following two parts: (1) power of data packet sent or received by the node represented by $\varpi_{R}$ and $\varpi_{T}$; and (2) power required for the lower power motoring operation represented by $\varpi_{LPL}^{x}$.

The main parameters of the WSN model adopted by this paper are similar to those of the X-MAC model, and the equipment limits are sourced from the internal data fragments of the prototype of the Thales sensor node [42]. Table 1 lists the values of all parameters.

### 3.5. Problem Statement

The main goal of this paper is to design a secure routing scheme using a traceback approach for EHWSNs that makes full use of available energy to ensure data integrity and improve data security. The approach can be characterized as follows:

(1) Data integrity. Assurance to the recipient of the data came from the expected sender and has not been altered in transit, although the data is sent to the sink after data aggregation and multi-hop routing.

(2) Maximizing the probability of successively routing the data packets to the sink. The probability of successively routing data to the sink can be defined as the ratio between the number of data packets received by the sink and the total number of data packets sent by the network. The maximum data routing success rate can be computed as follows:

(2) $$Max\left({ℬ}_{D}\right)=Max\left(\frac{{ℱ}_{r}}{{ℱ}_{t}}\right)$$

where ${ℱ}_{t}$ represents the total number of data packets sent in the network, and ${ℱ}_{r}$ represents the number of data packets successively received by the sink.

Moreover, notice messages reaching the sink also have a positive effect on network safety. They record the nodes that the routing path of data packets passes and then restore the routing path. If the sink receives the notice message but fails to receive the linked data packet or receive the altered data packet, it will find the malicious nodes attacked by tracing the source path of data packet through the notification with a high probability. Therefore, the TBSR scheme will also improve the success rate for notice messages to reach the sink:

(3) $$Max\left({ℬ}_{N}\right)=Max\left(\frac{A_{r}}{A_{t}}\right)$$

where $A_{t}$ represents the total number of notification sent in the network, and $A_{r}$ represents the number of notice messages received by the sink.

(3) Maximizing energy utilization

Energy utilization is the ratio of the energy consumed by the network to the available energy of the network within an hour, as shown in Equation (4):

(4) $$Max\left(C_{u}\right)=Max\left[\left(\overset{n}{\underset{i=1}{\sum }}{𝓌}_{i}\right)/\left(\overset{n}{\underset{i=1}{\sum }}{ℰ}_{ava}^{i}\right)\right]$$

where i is the i-th node in the network, n is the total number of nodes in the network, ${𝓌}_{i}$ represents the energy consumption of $n_{i}$ within an hour, and ${ℰ}_{ava}^{i}$ represents the available energy of $n_{i}$ within an hour, which is calculated according to Algorithm 1. The maximization of network energy utilization will improve the effective use of network energy so that the ratio of energy consumed to the available energy in the network is largest.

(4) Network lifetime 

In EHWSNs, the node will not die if the remaining battery level is maintained above 0 or a lower limit at any time.

(5) Minimizing demand for storage capacity of nodes

The storage capacity of a sensor node is limited, so the demand for storage capacity of nodes will not exceed the upper limit of the storage capacity of the sensor node. We assume that when the required storage capacity of node i in the strategy is ${𝓂}_{i}$, the maximum storage capacity required by the node is the smallest for the scheme, i.e., as follows:

(5) $$\min\left(ℳ\right)=\min\underset{0<i\leq n}{\max}\left({𝓂}_{i}\right)$$

(6) Minimizing convergence time Γ

Convergence time is the time taken by the information synchronization process after the router identifies the change of the topology structure of the network. Actually, when the victim is attacked, the attack path is reconstructed by consulting the information of upstream nodes and broadcasting malicious information.

(6) min(Γ)

In summary, the research objectives are as follows:

(7) $$\left\{ \begin{array}{c} Max\left({ℬ}_{D}\right)=Max\left(\frac{{ℱ}_{r}}{{ℱ}_{t}}\right)\\ Max\left({ℬ}_{N}\right)=Max\left(\frac{A_{r}}{A_{t}}\right)\\ Max\left(C_{u}\right)=Max\left[\left(\overset{n}{\underset{i=1}{\sum }}{𝓌}_{i}\right)/\left(\overset{n}{\underset{i=1}{\sum }}{ℰ}_{ava}^{i}\right)\right]\\ \min\left(ℳ\right)=\min\underset{0<i\leq n}{\max}\left({𝓂}_{i}\right)\\ \min\left(\Gamma \right) \end{array}\right.$$

## 4. TBSR Scheme Design

### 4.1. Research Motivation

The security problems of wireless sensor networks have been discussed for a long time and widely emphasized and researched in industrial and academic circles. The trust-based secure routing using traceback approach (TBSR) scheme in this paper is proposed to address the following problems concerning the secure routing of a network in the past research:

(1) The past multi-path routing schemes consume much energy and cannot ensure data integrity. The research objective of secure data collection is to ensure the monitoring data of sensor nodes can be routed to the sink safely. The attacker can appear at any position in the network, and the data packet can be attacked when it passes the area in which the attacker is located and then dropped. The principle of avoiding such attack is bypassing the area in which the attacker is located. However, the location of attacker cannot be determined in advance and bypassed. Therefore, most research adopts a multi-path or disjoint routing approach. The main feature of this approach is that multiple data packets are simultaneously sent to the destination through different routing paths, so although some routing paths are attacked, some data packets can reach the sink safely. The research [31] proposed the multi-path routing approach to defend against a selective forwarding attack. The multi-path routing approach sends multiple data packets through different routing paths. Thus, when the data packet on one path is attacked and dropped, the data packet can nonetheless reach the sink through other paths. Obviously, the multi-path scheme ensures data security to some extent. Nevertheless, the scheme has the disadvantage of sending one data packet multiple times, which increases energy consumption by a multiplier and seriously affects the network lifetime. Another important disadvantage of the scheme is that it cannot ensure data integrity. If the data packet is altered, it cannot be identified by the sink.
(2) The existing scheme to ensure the data integrity cannot avoid dropping of the data packet. Reference [59] proposed an ID-based aggregate signature scheme that can add a signature during data aggregation. The proposed scheme is able to ensure that the data packet with the signature can be authenticated, thereby ensuring data integrity. However, the scheme of adopting a digital signature cannot prevent the data packet from being dropped by the attacker.
(3) Although we proposed an Aggregate Signature-based Trust Routing scheme (ASTR) [58] that combines the digital signature and security data routing, the function of locating malicious nodes remains a requirement, so the scheme remains a positive secure defense approach. In ASTR scheme [58], the node sends ℳ data and N abstract packets (known as ℛ(ℳ,N) routing approach) to ensure both data routing security and data integrity. Despite high-energy consumption when the node sends ℳ data and N abstract packets, this research continues to lack the function to determine the position of malicious nodes.

Above all, how to design an active scheme to locate the malicious nodes and ensure data routing security and data integrity is a challenging issue. In this paper, we propose a scheme that integrates the traceback approach, adopts the ID-based aggregate signature method and routes data packets and notification through multiple paths. It both reduces the energy consumption and ensures the security and integrity of data. The TBSR scheme has the following features: (a) adopt the ID-based aggregate signature scheme to ensure the information can be authenticated; (b) multiple data packets and notification are generated simultaneously during the routing. The notification is used to determine whether the data packet has reached the destination safely and has the advantage of small size and low energy consumption; (c) the most important point is that it integrates the traceback scheme. The principle of the Traceback scheme to ensure security is to attach the ID number of nodes that data passes to the data packet when it is routed to the sink with a certain probability. This ID number information is called notification. Obviously, the more notification the sink receives, the more routing information of data packets will be contained in the notification when the network is attacked, so the amount of notification reflects the ability of the network to locate the malicious nodes. Therefore, in the traceback scheme, the probability of marking should be as high as possible. However, a higher probability of marking will increase the amount of notification and energy consumption of the network, which can affect the network lifetime. Its difference from the past traceback scheme is that EHWSNs can absorb solar energy, and the TBSR scheme cleverly designs the probability of marking and logging of nodes, which enable the scheme to make full use of the absorbed energy to improve network security. The scheme adopted by the TBSR is that when sensor nodes absorb sufficient energy, a high probability of marking and a low probability of logging are used. In this case, the sink can obtain more notification and improve network security. When nodes absorb less energy, for example at night, a low probability of marking and a high probability of logging can be used to store the notification on the nodes in the network instead of sending them to the sink immediately. In this case, when the network cannot absorb sufficient energy, a lesser amount of data can be transmitted in the network, which saves energy. When the battery on high level, the notification recorded on the nodes in the network by logging scheme will be sent to the sink. Overall, the scheme obviously improves system security and the availability of energy; (d) finally, the TBSR scheme uses the malicious node location function of the traceback to reduce the trust of malicious node and guides the data to bypass the nodes with low trust during the routing, which further improves the security of the system.

### 4.2. Trust-Based Secure Routing Scheme Design

**Figure 2:**
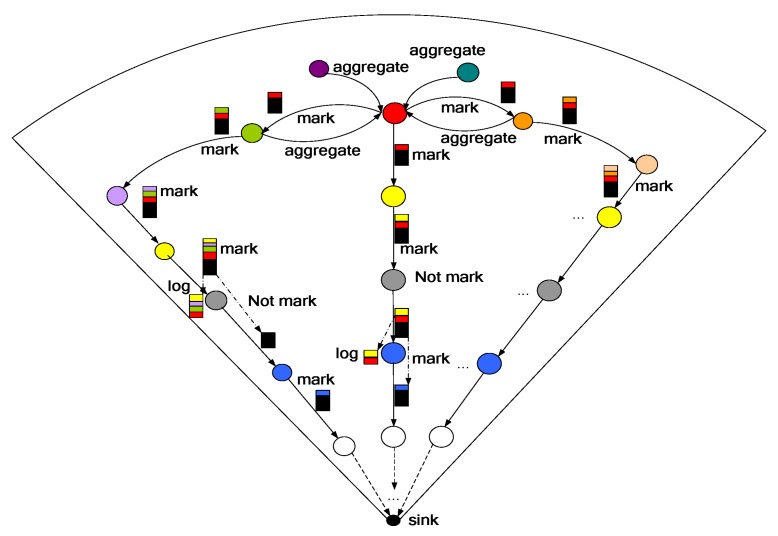
Framework of the TBSR scheme.

This section discusses the detailed design of the TBSR scheme. The TBSR scheme is shown in Figure 2. It is primarily composed of the following important parts: (1) data aggregate signature, (2) a data and notification disjoint routing approach, and (3) a traceback approach.

(1) aggregate signature stage

In this stage, ID-based aggregate signature technology [58] is adopted in the ASTR scheme ID-based aggregate signature can ensure the source nodes can send the data packets to the aggregator and the aggregator performs the aggregate signature and sends them to the sink after multiple hops, which can provide assurance to the recipient of the message came from the expected sender and has not been altered in transit [58]. Hence, in ASTR scheme, the data packets are not directly sent to the sink but sent after data aggregation, which effectively reduces the data amount loaded by nodes (see Figure 2). The process of data aggregation is shown in Figure 2. When the node $s_{0},\;s_{1},\;s_{2},\;s_{3},\;s_{4}$ intends to send the data packets to the sink, they will select one node among them, such as node $s_{0}$ as the aggregator while other nodes become the member nodes of aggregator node $s_{0}$ and send data packets to the aggregator node $s_{0}$. After receiving the data packets sent by all member nodes, the aggregator node $s_{0}$ adopts the aggregate signature scheme in Reference [58] to aggregate them into one data packet and sends the packet to the sink (if ℳ > 1, the data packet will be sent to the sink in a method similar to multi-path routing). Reference [58] has shown that the data aggregation method can be authenticated for each data of node. The selection of aggregator is similar to that of cluster head, which can be found in Reference [58].

(2) A data and notification disjoint routing approach

This section primarily discusses how to effectively route the data packet and notice message to the sink, i.e., a data and notification disjoint routing approach. ℳ data packets are sent each time using the multi-path routing scheme, and notification is generated for each data packet during the routing process through marking. Both data packet and notification are routed to the sink.

The procedure for this approach is as follows: first, an aggregator produces ℳ copies of the data packet during one operation and sends all copies to the sink through ℳ different paths. As shown in Figure 2, aggregator $s_{0}$ first generates a random number ${𝒹}_{i}$ in {1,𝒹}, and ${𝒹}_{i}$ represents the length of the i-th data packet routed horizontally before being routed to the sink with the shortest routing approach. In this paper, horizontal routing refers to each time the node selects a node on the left (right) that is the same hops as itself from the sink as the next relay node for routing. Thus, aggregator $s_{0}$ selects its neighbor node $s_{4}$ on the left as the relay node and sends the data packet to $s_{4}$. $s_{4}$ selects its neighbor node $s_{5}$ following the same direction. The process proceeds until the data packet is routed to node $s_{7}$, and the horizontal routing stops when its routing distance reaches ${𝒹}_{i}$. Starting from node $s_{7}$, the node will select the neighbor node closest to the sink until the data packet is routed to the sink. The routing process of other ℳ−1 data packets is the similar to the above. However, the difference is that the other ℳ−1 data packets will select the node that has not been selected by the preceding nodes or a highly trustable node as the relay node. The routing process of notification is very similar to the routing process of a data packet because the former is generated during the routing process of the data packet. The value of ℳ for routing of data packets is usually small, for example ℳ=2. 

(3) Traceback approach

The traceback approach primarily consists of two processes: marking and logging. In the TBSR scheme, the detailed description of the marking and logging process is as follows:

(a) Marking: For all data packets, before they reach the sink, the nodes generating the data packets and on the routing paths will be marked with a certain probability, and all nodes in the network are marked with the same probability at that time.
(b) Logging: Before reaching the sink, all data packets will be logged starting from the next hop destination of the source node with a certain probability, and all nodes in the network are logged in the same probability at a given time. The probability of marking and logging at each moment is determined by the current available power. The specific value should be calculated based on Algorithm 1:

**Algorithm 1.** the algorithm of obtaining available energy and obtaining the probability of marking and logging**INPUT: the observed solar radiation power** // $d_{i}\left(i\geq 0\right)$ is the i th day, $t_{i,j}\left(0\leq j\leq 23\right)$ is the j th hour of the i th day, // $F_{i,j}$ is the observed solar radiation power at $t_{i,j}$; $E_{initial}$ is the initial energy of the node battery, // $E_{max}$ is the max electricity in battery. **OUTPUT: the available energy** // $U_{i,j}$ is the available energy at $t_{i,j}$; $r_{i,j}$ is the remaining battery level. **(1) get available energy stage** **1:** Find a day with the minimum total observed solar radiation power in the whole day using the formula $sum_{i}=\int_{0}^{23}F_{i,j}\mathrm{dt}$. In addition, define this day as $d_{0}$. **2: If**
(i=0)     $t_{0,n}$ is the time to start the sunshine.     Get e using the formula $e=\lfloor \frac{E_{initial}}{n+1}\rfloor$.     Get $t_{0,𝕙}$ is the highest observed solar radiation time of the day.     **If**
(0≤j≤n)       $U_{0,0}=U_{0,1}=\cdots =U_{0,n}=e$;        $r_{0,j}=r_{0,j-1}+F_{0,j}-e$;}     **If**
(n+2≤j≤𝕙)       $U_{0,j}=F_{0,j}$;       $r_{0,j}=r_{0,n}$;     **If**
(𝕙+1≤j≤23)       $U_{0,13}=\cdots =U_{0,23}=e$;       $r_{0,j}=r_{0,j-1}+F_{0,j}-e$;     **If**
$\left(r_{i,j}\geq E_{max}\right)$       $r_{i,j}=E_{max}$;   **End if** **End if** **3: If**
(i≥1)     **Switch (**j**)**   **Case1:**     **If**
(0≤j≤n)
           $U_{i,0}=U_{i,1}=\cdots =U_{i,n}=e$;           $r_{i,j}=r_{i,j-1}+F_{i,j}-e$;     **Break;**       **Case2:**
     **If**
(j=n+1)           $U_{i,n+1}=U_{0,n+1}$;     **Break;**     **Case3:**     **If**
(n+2≤j<𝕙)           $U_{i,j}=F_{i,j-1}$;
          $r_{i,j}=r_{i,j-1}+F_{i,j}-U_{i,j}$;           **If**
$\left(r_{i,j}\geq E_{max}\right)$
           $r_{i,j}=E_{max}$;       **Break;**     **Case4:**
     **If**
(𝕙≤j≤𝕙+2)           $U_{i,j}=0.6F_{i,j-1}$;           $r_{i,j}=r_{i,j-1}+F_{i,j}-U_{i,j}$;           **If**
$\left(r_{i,j}\geq E_{max}\right)$
            $r_{i,j}=E_{max}$;     **Break;**     **Default:**
     **If**
(𝕙+2<j≤23)            $U_{i,0}=U_{i,1}=\cdots =U_{i,n}=e$;            $r_{i,j}=r_{i,j-1}+F_{i,j}-e$;            **If**
$\left(r_{i,j}\geq E_{max}\right)$
             $r_{i,j}=E_{max}$;     **Break;** **End if** **(2) get the probability of marking stage** **4: For** each $t_{i,j}$ in the set {$t_{i,0},t_{i,1},\ldots ,t_{i,23}$} **Do**     Get the probability of marking $\alpha_{i,j}$ using Equation (41); **End for** **(3) get the probability of logging stage** **5: For** each $t_{i,j}$ in the set {$t_{i,0},t_{i,1},\ldots ,t_{i,23}$} **Do**     Get the probability of logging $\beta_{i,j}$ using Equation (50); **End for**

The detailed description of the TBSR scheme is provided in Algorithm 2.

**Algorithm 2.** the algorithm of a trust-based secure routing (TBSR) scheme**INPUT: receive a packet** // $t_{i,j}\left(0\leq j\leq 23\right)$ is the j th hour of the i th day; $U_{i,j}$ is the available energy at $t_{i,j}$, // $\alpha_{i,j}$ is the probability of marking at $t_{i,j}$, $\beta_{i,j}$ is the probability of logging at $t_{i,j}$, and h is the hop from the sink. **OUTPUT: Forward a new packet to next hop node** **(1) aggregate signature stage** **1: For** each node **Do**     running aggregator determining algorithm which is similar to cluster-head selection algorithm in Reference [59]; **End for** // now, nodes either belong to aggregators or belong to member nodes **2: For** each member node **Do**    send its data and node ID, data time to its aggregator **End for** **3: For** each aggregator node $s_{0}$
**Do**     $s_{0}$ aggregate its member nodes’ data into a data packet $D_{0}$
  using ID-based aggregate signature technology as Reference [58];     $s_{0}$ aggregate its member nodes’ abstract into an abstract $A_{0}$  using ID-based aggregate signature technology as Reference [58]; **End for** **(2) Adopt the variable probability marking and logging**
$\left(\alpha_{i,j},\beta_{i,j}\right)$
**stage** **4: For** each receive packet P in node $n_{h}$ and $n_{h}$ is not sink **Do**     Mark all received packets P with $\alpha_{i,j}$.   //$\;\alpha_{i,j}$ using Equation (41). **End for** **5: For** each receive packet $P_{1}$ generated by last node $n_{h+1}$
**Do**     Log the amount of notification in packet $P_{1}$ with $\beta_{i,j}$.   //$\;\beta_{i,j}$ using Equation (50); **End for** **6:** Forward New packets $P^{\prime }$ to next hop node.

### 4.3. Optimized Selection of Parameters

In the TBSR scheme, the two most important parameters are probability of marking and probability of logging. The values of these two parameters are critical to the whole strategy. As the above shows, the solar radiation differs at different times, so the amount of energy that can be consumed by the nodes is different. The probabilities of marking and logging are calculated based on the available energy, so we should first calculate the amount of data received and sent by nodes, then calculate the energy consumption of data sending and receiving and finally makes the energy consumption less than available energy, obtaining satisfactory probabilities of marking and logging.

**Theorem** **1.** *For a planar network, assume the length of a data packet is* m *bits, the length of notification is* b *bits and the probability of marking is* α *. When the remaining battery level is low,* *the node sending the data packet will be logged with the probability of* β *after one hop, and the amount of data received and sent by the node that is* l *from the sink is represented with* ${𝕣}_{x}$ *and* ${𝕤}_{x}$ *respectively. Their calculation formulas are as follows:*

(8) $$\{\begin{array}{c} {𝕣}_{x}=\frac{\left(l+r\right)}{l}\times \left(m+b\alpha \right)p+\overset{z}{\underset{k=2}{\sum }}\frac{\left(l+kr\right)}{l}\times \left[mp^{k}+\left(1-\beta \right)b\alpha \overset{k}{\underset{i=1}{\sum }}p^{i}\right]|z=\lfloor \frac{R-l}{r}\rfloor \\ {𝕤}_{x}=\left(m+b\alpha \right)+\overset{z}{\underset{k=1}{\sum }}\frac{\left(l+kr\right)}{l}\times \left[mp^{k}+\left(1-\beta \right)b\alpha \overset{k}{\underset{i=0}{\sum }}p^{i}\right]|z=\lfloor \frac{R-l}{r}\rfloor \end{array}$$

**Figure 3:**
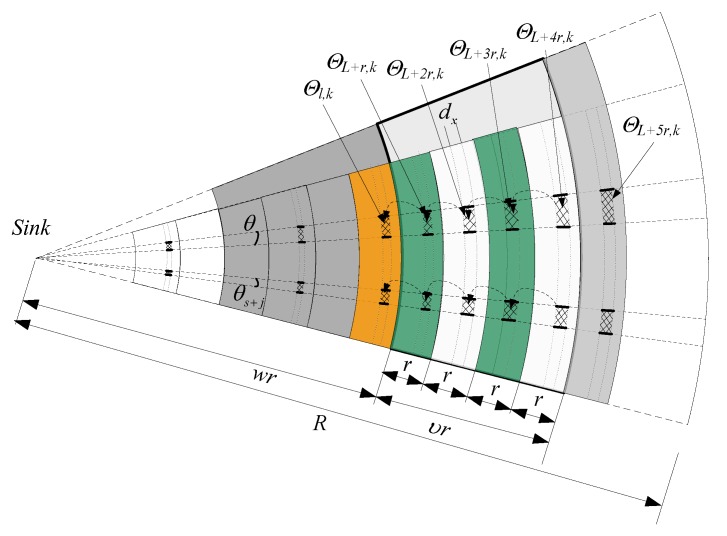
Illustration of the information loaded by a node.

**Proof.**  As shown in Figure 3, the node that is l from the sink is in the $\vartheta_{l,k}$ area with an angle of $\theta_{k}$. The emission radius of the node is r, so $\vartheta_{l,k}$ will surely receive the data generated in $\vartheta_{l+r,k}$ area that is r from itself. In the same manner, $\vartheta_{l+r,k}$ will receive and forward the data generated in the $\vartheta_{l+2r,k}$ area. If the $\vartheta_{l,k}$ area is very small, all nodes in the area can be considered loading the same amount of data. The amount of data received by the node $n_{x}$ that is l from the sink is represented by ${\mathbb{R}}_{x}$.

The inclusion angle between the $\vartheta_{l,k}$ area and the sink is as small as $d_{\theta k}$ (arc), the width is assumed $d_{x},\;$and the $\vartheta_{l,k}$ area is fan-shaped. However, the width is small, so in differential calculus, it can be considered a rectangle for area calculation; the length is equal to the arc length, i.e., the width of $d_{\theta k}l$ is $d_{x}$. Therefore, the area of $\vartheta_{l+r,k}$ is $S_{\vartheta_{l+r,k}}=\left(l+r\right)d_{\theta k}d_{x}$. The total number of nodes in the $\;\vartheta_{l+r,k}$ area is as follows:

(9) $$N_{\vartheta_{l+r,k}}=S_{\vartheta_{l+r,k}}\rho =\rho \left(l+r\right)d_{\theta k}d_{x}$$

The length of marking position is b bits, so when a data packet is sent from the $\vartheta_{l+r,k}$ area to the $\vartheta_{l,k}$ area, the length of the data packet is as follows:

(10) $$v_{\vartheta_{l+r,k}}=p\left(m+b\alpha \right)$$

The $N_{\vartheta_{l+r,k}}$ nodes in the $\vartheta_{l+r,k}$ will surely generate $N_{\vartheta_{l+r,k}}$ data packets. Therefore, all data packets in the $\vartheta_{l+r,k}$ area will be transmitted to the $\vartheta_{l,k}$ area. The amount of data at this moment is as follows:

(11) $$\gamma_{\vartheta_{l+r,k}}=\rho \left(l+r\right){𝑑}_{\theta k}d_{x}\times \left(m+b\alpha \right)p$$

The area and number of nodes in the $\vartheta_{l+2r,k}$ area are as follows:

(12) $$S_{\vartheta_{l+2r,k}}=\left(l+2r\right)d_{\theta k}d_{x},\;N_{\vartheta_{l+2r,k}}=S_{\vartheta_{l+2r,k}}\rho =\rho \left(l+2r\right)d_{\theta k}d_{x}$$

When a data packet is transmitted from the $\vartheta_{l+2r,k}$ area to the $\vartheta_{l,k}$ area, the length of data packet should be as follows:

(13) $$v_{\vartheta_{l+2r,k}}=\beta mp^{2}+\left(1-\beta \right)\times \left[\left(m+b\alpha \right)p^{2}+b\alpha p\right]$$

Similarly, when all data packets are sent from the $\vartheta_{l+2r,k}$ area to the $\vartheta_{l,k}$ area, the amount of data should be as follows:

(14) $$\gamma_{\vartheta_{l+2r,k}}=\rho \left(l+2r\right)d_{\theta k}d_{x}\times \left\{\beta mp^{2}+\left(1-\beta \right)\times \left[\left(m+b\alpha \right)p^{2}+b\alpha p\right]\right\}$$

At this time, the required storage space of each node in the $\vartheta_{l+r,k}$ area is as follows:

(15) $${ℵ}_{l+r}=b\alpha p\;or\;0$$

The area and number of nodes of the $\vartheta_{l+3r,k}$ area are as follows:

(16) $$S_{\vartheta_{l+3r,k}}=\left(l+3r\right)d_{\theta k}d_{x},N_{\vartheta_{l+3r,k}}=S_{\vartheta_{l+3r,k}}\rho =\rho \left(l+3r\right)d_{\theta k}d_{x}$$

When a data packet is transmitted from the $\vartheta_{l+3r,k}$ area to the $\vartheta_{l,k}$ area, the total length of the data packet should be the following:

(17) $$v_{\vartheta_{l+3r,k}}=\beta mp^{3}+\left(1-\beta \right)\times \left[\left(m+b\alpha \right)p^{3}+b\alpha p^{2}+b\alpha p\right]$$

Similarly, when all data packets are transmitted from the $\vartheta_{l+3r,k}$ area to the $\vartheta_{l,k}$ area, the amount of data should be as follows:

(18) $$\gamma_{\vartheta_{l+3r,k}}=\rho \left(l+3r\right)d_{\theta k}d_{x}\times \left\{\beta mp^{3}+\left(1-\beta \right)\times \left[\left(m+b\alpha \right)p^{3}+b\alpha p^{2}+b\alpha p\right]\right\}$$

At this time, the storage space of each node in the $\vartheta_{l+2r,k}$ area is as follows:

(19) $${ℵ}_{l+2r}=b\alpha p\;or\;0$$

Similarly, when all data packets are transmitted from the $\vartheta_{l+zr,k}$ to the $\vartheta_{l,k}$, the amount of data should be the following:

(20) $$\gamma_{\vartheta_{l+zr,k}}=\rho \left(l+zr\right)d_{\theta k}d_{x}\times \left\{\beta mp^{z}+\left(1-\beta \right)\times \left[\left(m+b\alpha \right)p^{z}+\cdots +b\alpha p^{2}+b\alpha p\right]\right\}$$

At this time, the storage space of each node in the $\vartheta_{l+\left(z-1\right)r,k}$ area equal the following:

(21) $${ℵ}_{l+\left(z-1\right)r}=b\alpha p\;or\;0$$

Similarly, the amount of data received by the $\vartheta_{l,k}$ area can be calculated as follows:

(22) $$\begin{array}{ll} {𝕣}_{\vartheta_{l,k}}=\gamma_{\vartheta_{l+r,k}}+ & \gamma_{\vartheta_{l+2r,k}}+\cdots +\gamma_{\vartheta_{l+zr,k}}\\ & =\rho \left(l+r\right)d_{\theta k}d_{x}\times \left(m+b\alpha \right)p+\rho \left(l+2r\right)d_{\theta k}d_{x}\\ & \times \left\{\beta mp^{2}+\left(1-\beta \right)\times \left[\left(m+b\alpha \right)p^{2}+b\alpha p\right]\right\}+\cdots +\rho \left(l+zr\right)d_{\theta k}d_{x}\\ & \times \left\{\beta mp^{z}+\left(1-\beta \right)\times \left[\left(m+b\alpha \right)p^{z}+\cdots +b\alpha p^{2}+b\alpha p\right]\right\}\\ & =\rho d_{\theta k}d_{x}\{\left(l+r\right)\times \left(m+b\alpha \right)p\\ & +\overset{z}{\underset{k=2}{\sum }}\left(l+kr\right)\times \left[mp^{k}+\left(1-\beta \right)b\alpha \overset{k}{\underset{i=1}{\sum }}p^{i}]\right\} \end{array}$$

Thus, the amount of data received by each node in the $\vartheta_{l,k}$ area can be calculated as follows:

(23) 𝕣x=𝕣ϑl,kρldθkdx      =(l+r)l×(m+bα)p      +∑k=2z(l+kr)l×[mpk+(1−β)bα∑i=1kpi]|z=⌊R−lr⌋

The next step is calculating the amount of data sent by nodes. The length of data packet sent from the $\vartheta_{l,k}$ area is as follows:

(24) $$\omega_{\vartheta_{l,k}}=m+b\alpha$$

The $N_{\vartheta_{l,k}}$ nodes in the $\vartheta_{l,k}$ will surely generate $N_{\vartheta_{l,k}}$ data packets, so the amount of data sent from the $\vartheta_{l,k}$ area is as follows:

(25) $$L_{\vartheta_{l,k}}=\rho ld_{\theta k}d_{x}\left(m+b\alpha \right)$$

When a data packet is transmitted from the $\vartheta_{l+r,k}$ area to the $\vartheta_{l,k}$ and sent out by the $\vartheta_{l,k}$ area, the length of data packet should be as follows:

(26) $$\omega_{\vartheta_{l+r,k}}=\beta mp+\left(1-\beta \right)\times \left[\left(m+b\alpha \right)p+b\alpha \right]$$

The total number of nodes in the abovementioned $\vartheta_{l+r,k}$ area, i.e., total number of generated data packets is $N_{\vartheta_{l+r,k}}=\rho \left(l+r\right)d_{\theta k}d_{x}$. Therefore, when all data packets are transmitted from the $\vartheta_{l+r,k}$ area to the $\vartheta_{l,k}$ area, the amount of data should be the following:

(27) $$L_{\vartheta_{l+r,k}}=\rho \left(l+r\right)d_{\theta k}d_{x}\times \left\{\beta mp+\left(1-\beta \right)\cdot \left[\left(m+b\alpha \right)p+b\alpha \right]\right\}$$

Similarly, when a data packet is sent from the $\vartheta_{l+2r,k}$ area to the $\vartheta_{l,k}$ area, the length of data packet at this moment should be the following:

(28) $$\omega_{\vartheta_{l+2r,k}}=\beta mp^{2}+\left(1-\beta \right)\times \left[\left(m+b\alpha \right)p^{2}+b\alpha p+b\alpha \right]$$

The number of data packets produced by the $\vartheta_{l+2r,k}$ area is $N_{\vartheta_{l+2r,k}}=\rho \left(l+2r\right)d_{\theta k}d_{x}$. Therefore, when all data packets are sent from the $\vartheta_{l+2r,k}$ area to the $\vartheta_{l,k}$, the amount of data sent out from the area should be the following:

(29) $$L_{\vartheta_{l+2r,k}}=\rho \left(l+2r\right)d_{\theta k}d_{x}\times \left\{\beta mp^{2}+\left(1-\beta \right)\times \left[\left(m+b\alpha \right)p^{2}+b\alpha p+b\alpha \right]\right\}$$

Similarly, when a data packet is sent from the $\vartheta_{l+zr,k}$ area to the $\vartheta_{l,k}$ area, the length of the data packet should be as follows:

(30) $$\omega_{\vartheta_{l+zr,k}}=\beta mp^{z}+\left(1-\beta \right)\times \left[\left(m+b\alpha \right)p^{z}+\cdots +b\alpha p^{2}+b\alpha p+b\alpha \right]$$

The number of data packets generated in the $\vartheta_{l+zr,k}$ area is $N_{\vartheta_{l+zr,k}}=\rho \left(l+zr\right)d_{\theta k}d_{x}$. Therefore, when all data packets are transmitted from the $\vartheta_{l+zr,k}$ area to the $\vartheta_{l,k}$, the amount of data sent out from the area should be the following:

(31) $$\begin{array}{l} L_{\vartheta_{l+zr,k}}=\rho \left(l+zr\right)d_{\theta k}d_{x}\\ \;\times \left\{\beta mp^{z}+\left(1-\beta \right)\times \left[\left(m+b\alpha \right)p^{z}+\cdots +b\alpha p^{2}+b\alpha p+b\alpha \right]\right\} \end{array}$$

Calculated in the same manner, the amount of data sent out from the $\vartheta_{l,k}$ area is as follows:

(32) $$\begin{array}{ll} {𝕤}_{\vartheta_{l,k}}=L_{\vartheta_{l,k}}+ & L_{\vartheta_{l+r,k}}+L_{\vartheta_{l+2r,k}}+\cdots +L_{\vartheta_{l+zr,k}}\\ & =\rho ld_{\theta k}d_{x}\left(m+b\alpha \right)+\rho \left(l+r\right)d_{\theta k}d_{x}\\ & \times \left\{\beta mp+\left(1-\beta \right)\times \left[\left(m+b\alpha \right)p+b\alpha \right]\right\}+\rho \left(l+2r\right)d_{\theta k}d_{x}\\ & \times \left\{\beta mp^{2}+\left(1-\beta \right)\times \left[\left(m+b\alpha \right)p^{2}+b\alpha p+b\alpha \right]\right\}+\cdots \\ & +\rho \left(l+zr\right)d_{\theta k}d_{x}\\ & \times \left\{\beta mp^{z}+\left(1-\beta \right)\times \left[\left(m+b\alpha \right)p^{z}+\cdots +b\alpha p^{2}+b\alpha p+b\alpha \right]\right\}\\ & =\rho d_{\theta k}d_{x}\left\{l\left(m+b\alpha \right)+\overset{z}{\underset{k=1}{\sum }}\left(l+kr\right)\times \left[mp^{k}+\left(1-\beta \right)b\alpha \overset{k}{\underset{i=0}{\sum }}p^{i}\right]\right\} \end{array}$$

Thus, the amount of data sent by each node in the $\vartheta_{l,k}$ is as follows:

(33) 𝕤x=𝕤ϑl,kρldθkdx=(m+bα)+Σk=1z(l+kr)l×[mpk+(1−β)bαΣi=0kpi]|z=⌊R−lr⌋

□

**Theorem** **2.** *This paper adopts the simplified X-MAC energy protocol. Thus, the energy consumption of a node* $\varpi_{tot}^{x}$ *has two parts: (1) power of data packet sent or received by the node represented by* $\varpi_{R}$ *and* $\varpi_{T}$*; and (2)* *power required for the lower power motoring operation represented by* $\varpi_{LPL}^{x}$*. Assuming* $\varpi_{tot}^{x}$ *represents total energy consumption of communication and Low-Power Listening of the node that is* x *m from the sink in one communication period* $t_{com}$*,* $\varpi_{LPL}^{x}$ *represents the energy required for LPL operation,* $\varpi_{R}^{x}$ *represents the power consumed when one node receives one data packet,* $\varpi_{T}^{x}$ *represents the power consumption of sending one data packet, and* $\delta_{r}^{x}$ *and* $\delta_{t}^{x}$ *represent the amount of data received and sent by one node. After this paper simplifies the energy consumption of the perception stage,* $\varpi_{tot}^{x}$ *can be calculated by the following formula*:

(34) $$\varpi_{\mathrm{tot}}^{x}=\varpi_{\mathrm{LPL}}^{x}+\varpi_{R}^{x}\delta_{r}^{x}+\varpi_{T}^{x}\delta_{t}^{x}$$

*where*

(35) $$\{\begin{array}{c} \varpi_{T}^{x}={𝓅}_{t}T_{d}+\frac{\left(1-D_{\mathrm{com}}\right)t_{\mathrm{com}}}{2\left(T_{p}+T_{\mathrm{al}}\right)}\left({𝓅}_{t}T_{p}+{𝓅}_{r}T_{\mathrm{al}}\right)\\ \varpi_{R}^{x}={𝓅}_{r}T_{d}+\left({𝓅}_{r}T_{p}+{𝓅}_{t}T_{\mathrm{al}}\right)\\ \varpi_{\mathrm{LPL}}^{x}={𝓅}_{r}D_{\mathrm{com}}+{𝓅}_{t}\left(1-D_{\mathrm{com}}\right)-\pi_{t}^{x}-\pi_{r}^{x}\\ \pi_{t}^{x}=\left\{{𝓅}_{s}\left[\frac{\left(1-D_{\mathrm{com}}\right)t_{\mathrm{com}}}{2}+T_{p}+T_{\mathrm{al}}\right]+{𝓅}_{r}T_{p}\right\}\frac{\delta_{t}^{x}}{t_{\mathrm{com}}}\\ \pi_{r}^{x}=\left[(T_{\mathrm{al}}+T_{d}){𝓅}_{s}+{𝓅}_{r}T_{p}\right]\frac{\delta_{r}^{x}}{t_{\mathrm{com}}} \end{array}$$

**Proof.**  According to the X-MAC energy consumption model, the average energy consumption of sending one data packet $\varpi_{T}^{x}$ includes two parts—the energy consumption of sending the data part of the data packet and the energy consumption of a periodic preface transmission to notify the receiving node that a data packet will reach. Therefore, the average energy consumption of sending one data packet $\varpi_{T}^{x}$ can be calculated by the following formula:

(36) $$\varpi_{T}^{x}={𝓅}_{t}T_{d}+\frac{\left(1-D_{\mathrm{com}}\right)t_{\mathrm{com}}}{2\left(T_{p}+T_{\mathrm{al}}\right)}\left({𝓅}_{t}T_{p}+{𝓅}_{r}T_{\mathrm{al}}\right)$$

According to the X-MAC energy consumption model, the average energy consumption of receiving one data packet $\varpi_{R}^{x}$ can be calculated by the following formula:

(37) $$\varpi_{R}^{x}={𝓅}_{r}T_{d}+\left({𝓅}_{r}T_{p}+{𝓅}_{t}T_{\mathrm{al}}\right)$$

The corresponding power of LPL operation can be calculated as follows:

(38) $$\varpi_{\mathrm{LPL}}^{x}={𝓅}_{r}D_{\mathrm{com}}+{𝓅}_{t}\left(1-D_{\mathrm{com}}\right)-\pi_{t}^{x}-\pi_{r}^{x}$$

The reason for deducting $\pi_{t}^{x}$ and $\pi_{r}^{x}$ from $\varpi_{\mathrm{LPL}}^{x}$ is that when the node is in active status, some time is spent on sending and receiving data and has been calculated by Equations (36) and (37), so the energy consumption during this period should be deducted in the calculation of the energy consumption of LPL operation. Obviously, the nodes closer to the sink load mode data, so they spend more time on sending and receiving data and less time on LPL operation, i.e., the deducted part $\pi_{t}^{x}$ and $\pi_{r}^{x}$ are larger and $\varpi_{\mathrm{LPL}}^{x}$ is smaller. In contrast, the nodes far from the sink node load less data, so they spend a long time on LPL operation, i.e., $\pi_{t}^{x}$ and $\pi_{r}^{x}$ are smaller and $\varpi_{\mathrm{LPL}}^{x}$ is larger.

According to the X-MAC energy consumption model, $\pi_{t}^{x}$ can be calculated as follows:

(39) $$\pi_{t}^{x}=\left\{{𝓅}_{s}\left[\frac{\left(1-D_{\mathrm{com}}\right)t_{\mathrm{com}}}{2}+T_{p}+T_{\mathrm{al}}\right]+{𝓅}_{r}T_{p}\right\}\frac{\delta_{t}^{x}}{t_{\mathrm{com}}}$$

$\pi_{r}^{x}$ can be calculated as follows:

(40) $$\pi_{r}^{x}=\left[(T_{\mathrm{al}}+T_{d}){𝓅}_{s}+{𝓅}_{r}T_{p}\right]\frac{\delta_{r}^{x}}{t_{\mathrm{com}}}$$

**Figure 4:**
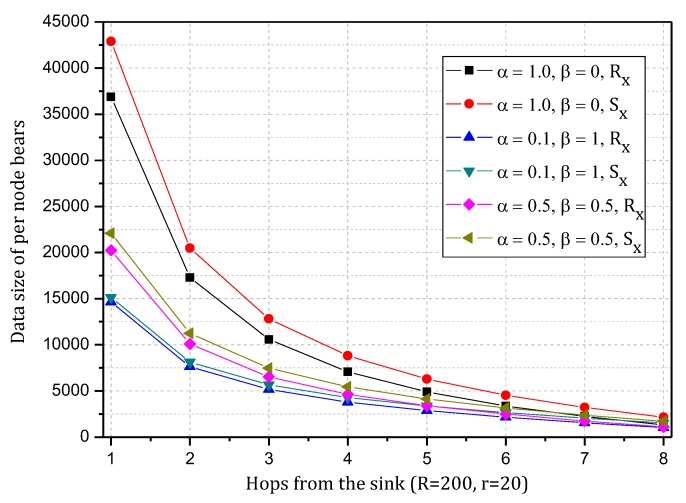
Amount of data received and sent by nodes—different hops from the sink under different marking and logging probabilities.

Figure 4 shows the amount of data received and sent by nodes in the network in the TBSR scheme. As the figure shows, when α=1.0, β=0, i.e., the probability of marking is 1 and the probability of logging is 0, the amount of data received and sent is greatly different from that in other cases. When h=1, the amount of received data is 1.82 times the amount when α=0.5, β=0.5 and 2.52 times the amount when α=1.0, β=0. In the latter case, all nodes on the path are marked and not stored, which achieves the best security. If the network is attacked, all source nodes sending data packets and nodes on the transmission path can be found. If the node battery level is high, this case (α=1.0, β=0) has the highest security. However, if the level of battery remaining is low, the probability of marking should be lowered and the probability of logging should be improved to ensure the smooth transmission of data and avoid the death of a node, which will save energy due to smaller amounts of received and sent data.

**Figure 5:**
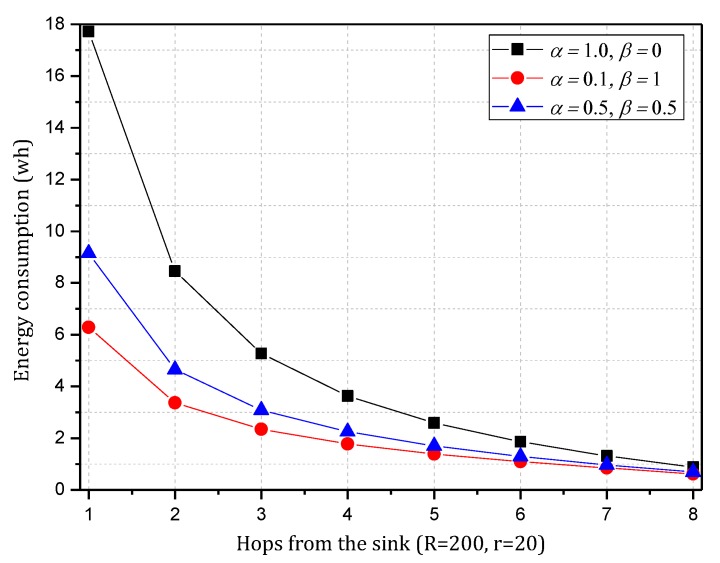
Energy consumption of nodes—different hops from the sink for receiving and sending data under different probability of marking and logging.

Figure 5 shows the analysis of the node h = 1. When α=1.0, β=0, the electrical energy consumed by the node for receiving and sending data is 17.71 wh. When α=0.5, β=0.5, the electrical energy consumed by the node for receiving and sending data is approximately 9.15 wh. When α=0.1, β=1, the consumed electrical energy is only 6.29 wh. The last one saves 64.48% and 31.26% energy, respectively, compared with the first and second case. Solar radiation and the electrical energy compensated for the battery per hour varies under different climate conditions and environmental factors. In order to maintain the level of battery remaining above 0 or a lower limit at any time, the level of battery remaining determines the values of α and β we can use. As shown in Figure 5, we can adjust the probability of marking α and probability of logging β to achieve different energy consumptions, thereby adapting to different climate conditions and environmental factors.

**Figure 6:**
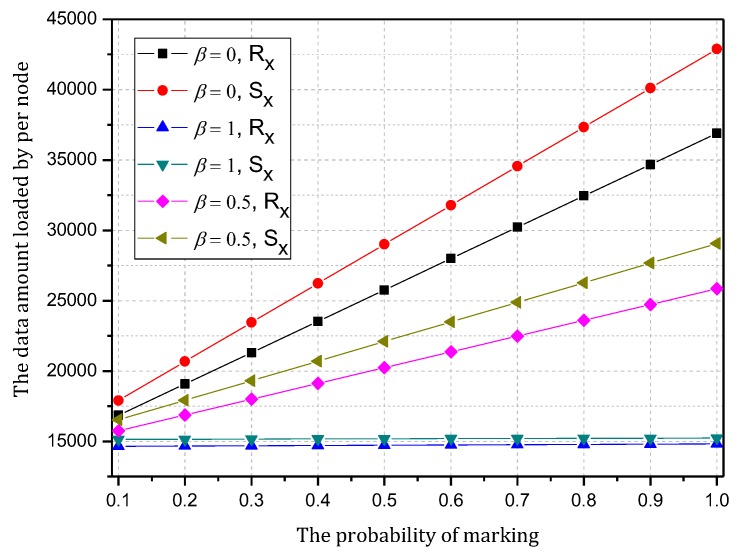
Amount of data received and sent—a node 1 hop from the sink under different probability of marking and logging.

Figure 6 shows the amount of data received and sent by the node 1 hop from the sink when the probability of marking is 0.1~1 and the probability of logging is 0, 0.5 and 1. As the figure shows, when the probability of logging is the same, the amount of data and probability of marking present a positive linear correlation, so the greater the probability of marking is, the more data the node will load. In addition, a greater probability of logging results in a greater slope and faster increase in data amount.

**Figure 7:**
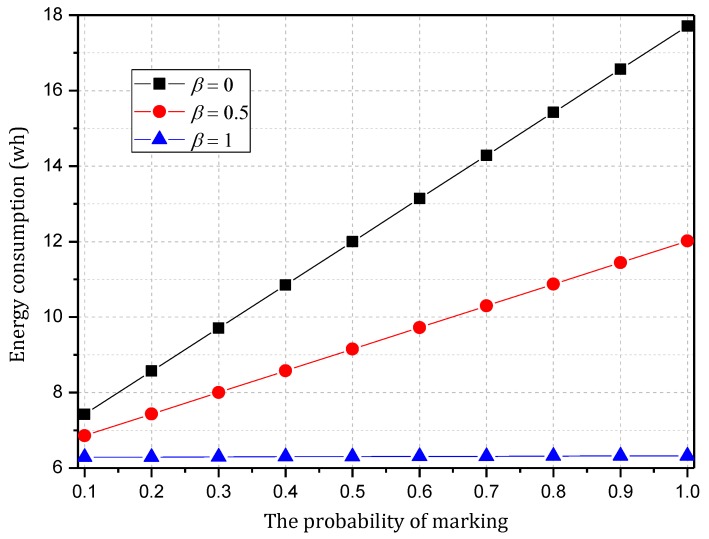
Energy consumption of the node 1 hop from the sink for receiving and sending data under different probability of marking and logging.

Figure 7 shows the energy consumption of the node under the conditions provided in Figure 6. As the figure shows, when β=1, the energy consumption changes insignificantly as α increases and maintains approximately 6.30 wh. In this case, although the energy consumption is small, the probability of logging is 1, i.e., the mark will be stored after the next hop, and all subsequent nodes on the routing path cannot be marked, so network security is very low. When β=0.5, the minimum energy consumption is approximately 7 wh and the maximum is approximately 12 wh. When β=0, the maximum energy consumption can approach 18 wh. If the energy is sufficient, this case will have the highest security. In conclusion, increasing α can improve network security, and increasing β will reduce network security, so we must determine proper values for α and β to save energy while ensuring higher security.

**Figure 8:**
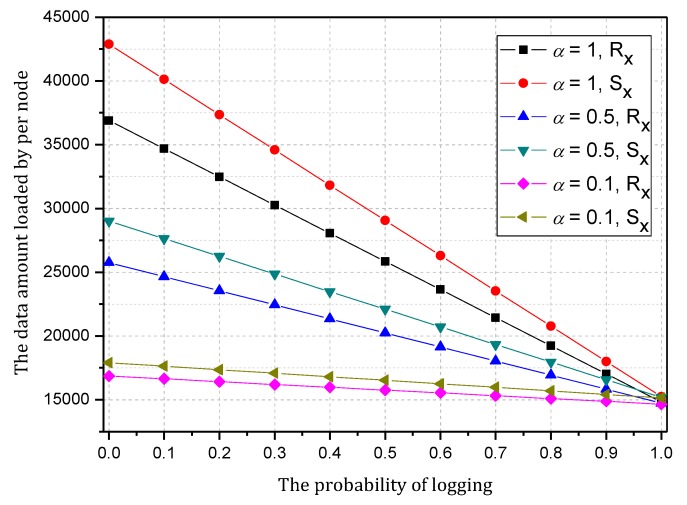
Amount of data received and sent—a node 1 hop from the sink under different logging and probability of marking.

**Figure 9:**
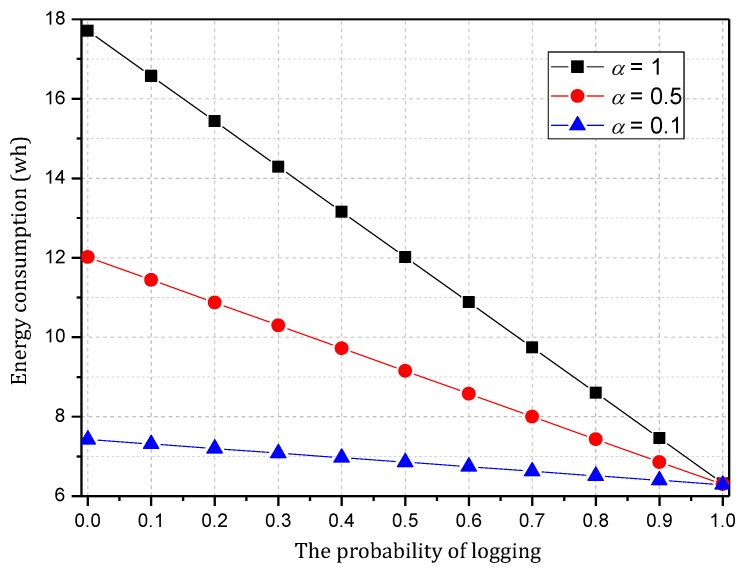
Energy consumption of the node 1 hop from the sink for receiving and sending data under different logging and probability of marking.

Figure 8 shows the amount of data received and sent by the node 1 hop from the sink when the probability of logging is 0~1 and the probability of marking is 0, 0.5 and 1. As the figure shows, when the probability of marking is the same, the amount of data and probability of logging present a negative linear correlation. Thus, the greater the probability of logging is, the less data the node will load. In addition, a greater probability of logging results in a greater absolute value of slope and faster decrease of data amount. Figure 9 shows the energy consumption of the node under the conditions provided in Figure 8. As the figure shows, when α=0.1, the energy consumption changes insignificantly as β increases and maintains within 6.29~7.42 wh.

The TBSR scheme can determine the proper probability of marking and logging based on the level of battery remaining and solar radiation, as seen in Figure 7 and Figure 9. Therefore, we should do further research to obtain the maximum α and minimum β under the same energy consumption.

**Theorem** **3.** *For a planar network, assume that the length of data packet is* m *bits and the length of notification is* b *bits. The node sending the data packet will be logged with the probability of* β *after one hop, the amount of data received and sent by the node that is* l *from the sink is represented with* ${𝕣}_{x}$ *and* ${𝕤}_{x}$ *respectively, the energy consumption of each node is* $W_{tot}$*, and the energy supplied by the battery during the period is* U*. To ensure the energy consumption is less than or equal to the energy supplied,* i.e., $W_{tot}\leq U$*, the probability of marking shall meet the following conditions*:

(41) $$\alpha \leq \frac{U-\nu_{1}\left(\varpi_{R}^{x}-f\right)-\nu_{2}\left(\varpi_{T}^{x}-g\right)-c}{\mu_{1}\left(\varpi_{R}^{x}-f\right)+\mu_{2}\left(\varpi_{T}^{x}-g\right)},when\;\beta \;\mathrm{is}\;a\;\mathrm{fixed}\;\mathrm{value}$$

*where*

(42) $$\{\begin{array}{c} \varpi_{R}^{x}={𝓅}_{r}T_{d}+\left({𝓅}_{r}T_{p}+{𝓅}_{t}T_{\mathrm{al}}\right)\\ \varpi_{T}^{x}={𝓅}_{t}T_{d}+\frac{\left(1-D_{\mathrm{com}}\right)t_{\mathrm{com}}}{2\left(T_{p}+T_{\mathrm{al}}\right)}\left({𝓅}_{t}T_{p}+{𝓅}_{r}T_{\mathrm{al}}\right)\\ c={𝓅}_{r}D_{\mathrm{com}}+{𝓅}_{t}\left(1-D_{\mathrm{com}}\right)\\ f=\frac{\left[(T_{\mathrm{al}}+T_{d}){𝓅}_{s}+{𝓅}_{r}T_{p}\right]}{t_{\mathrm{com}}}\\ g=\frac{\left\{{𝓅}_{s}\left[\frac{\left(1-D_{\mathrm{com}}\right)t_{\mathrm{com}}}{2}+T_{p}+T_{\mathrm{al}}\right]+{𝓅}_{r}T_{p}\right\}}{t_{\mathrm{com}}}\\ \mu_{1}=\frac{l+r}{l}bp+\overset{z}{\underset{k=2}{\sum }}\frac{l+kr}{l}\left(1-\beta \right)b\times \overset{k}{\underset{i=1}{\sum }}p^{i}\\ \mu_{2}=b+\overset{z}{\underset{k=1}{\sum }}\frac{l+kr}{l}\left(1-\beta \right)b\times \overset{k}{\underset{i=0}{\sum }}p^{i}\\ \nu_{1}=\frac{l+r}{l}m+\overset{z}{\underset{k=2}{\sum }}\frac{l+kr}{l}mp^{k}\\ \nu_{2}=m+\overset{z}{\underset{k=1}{\sum }}\frac{l+kr}{l}mp^{k} \end{array}$$

**Proof.**  The formula is obtained based on the energy consumption: $W_{\mathrm{tot}}=\varpi_{\mathrm{LPL}}^{x}+\varpi_{R}^{x}{𝕣}_{x}+\varpi_{T}^{x}{𝕤}_{x}$. To ensure that the energy consumption is less than the supplied energy, i.e., $W_{\mathrm{tot}}\leq U$, the following formula is obtained:

$${𝓅}_{r}D_{\mathrm{com}}+{𝓅}_{t}\left(1-D_{\mathrm{com}}\right)-\left\{{𝓅}_{s}\left[\frac{\left(1-D_{\mathrm{com}}\right)t_{\mathrm{com}}}{2}+T_{p}+T_{\mathrm{al}}\right]+{𝓅}_{r}T_{p}\right\}\frac{{𝕤}_{x}}{t_{\mathrm{com}}}\;-\left[(T_{\mathrm{al}}+T_{d}){𝓅}_{s}+{𝓅}_{r}T_{p}\right]\frac{{𝕣}_{x}}{t_{\mathrm{com}}}+\varpi_{R}^{x}{𝕣}_{x}+\varpi_{T}^{x}{𝕤}_{x}\leq E$$

Let $c={𝓅}_{r}D_{\mathrm{com}}+{𝓅}_{t}\left(1-D_{\mathrm{com}}\right)$, $f=\frac{\left[(T_{\mathrm{al}}+T_{d}){𝓅}_{s}+{𝓅}_{r}T_{p}\right]}{t_{\mathrm{com}}}$, $g=\frac{\left\{{𝓅}_{s}\left[\frac{\left(1-D_{\mathrm{com}}\right)t_{\mathrm{com}}}{2}+T_{p}+T_{\mathrm{al}}\right]+{𝓅}_{r}T_{p}\right\}}{t_{\mathrm{com}}}$, 

Then:

(43) $$c+\left(\varpi_{R}^{x}-f\right){𝕣}_{x}+\left(\varpi_{T}^{x}-g\right){𝕤}_{x}\leq U$$

According to Theorem 1:

(44) $${𝕣}_{x}=\frac{\left(l+r\right)}{l}\times \left(m+b\alpha \right)p+\overset{z}{\underset{k=2}{\sum }}\frac{\left(l+kr\right)}{l}\times \left[mp^{k}+\left(1-\beta \right)b\alpha \overset{k}{\underset{i=1}{\sum }}p^{i}\right]$$

(45) $${𝕤}_{x}=\left(m+b\alpha \right)+\overset{z}{\underset{k=1}{\sum }}\frac{\left(l+kr\right)}{l}\times \left[mp^{k}+\left(1-\beta \right)b\alpha \overset{k}{\underset{i=0}{\sum }}p^{i}\right]$$

Treat β as a fixed value and transpose Equation (44):

(46) $${𝕣}_{x}=\left[\frac{l+r}{l}bp+\overset{z}{\underset{k=2}{\sum }}\frac{l+kr}{l}\left(1-\beta \right)b\times \overset{k}{\underset{i=1}{\sum }}p^{i}\right]\times \alpha +\frac{l+r}{l}m+\overset{z}{\underset{k=2}{\sum }}\frac{l+kr}{l}mp^{k}$$

Let $\mu_{1}=\frac{l+r}{l}bp+\Sigma_{k=2}^{z}\frac{l+kr}{l}\left(1-\beta \right)b\times \Sigma_{i=1}^{k}p^{i}$, $\nu_{1}=\frac{l+r}{l}m+\Sigma_{k=2}^{z}\frac{l+kr}{l}mp^{k}$.

Transpose Equation (45):

(47) $${𝕤}_{x}=\left[b+\overset{z}{\underset{k=1}{\sum }}\frac{l+kr}{l}\left(1-\beta \right)b\times \overset{k}{\underset{i=0}{\sum }}p^{i}\right]\times \alpha +m+\overset{z}{\underset{k=1}{\sum }}\frac{l+kr}{l}mp^{k}$$

Let $\mu_{2}=b+\Sigma_{k=1}^{z}\frac{l+kr}{l}\left(1-\beta \right)b\times \Sigma_{i=0}^{k}p^{i}$, $\nu_{2}=m+\Sigma_{k=1}^{z}\frac{l+kr}{l}mp^{k}$.

Substitute ${𝕣}_{x}=\mu_{1}\alpha +\nu_{1}$ and ${𝕤}_{x}=\mu_{2}\alpha +\nu_{2}$ in Equation (43):

(48) $$c+\left(\varpi_{R}^{x}-f\right)\left(\mu_{1}\alpha +\nu_{1}\right)+\left(\varpi_{T}^{x}-g\right)\left(\mu_{2}\alpha +\nu_{2}\right)\leq U$$

Transpose:

(49) $$\alpha \leq \frac{U-\nu_{1}\left(\varpi_{R}^{x}-f\right)-\nu_{2}\left(\varpi_{T}^{x}-g\right)-c}{\mu_{1}\left(\varpi_{R}^{x}-f\right)+\mu_{2}\left(\varpi_{T}^{x}-g\right)}$$

□

**Theorem** **4.** *For a planar network, assume that the length of data packet is* m *bits and the length of notification is* b *bits.* *The node sending the data packet will be marked in the probability of* α*, the amount of data received and sent by the node that is* l *from the sink is represented with* ${𝕣}_{x}$ *and* ${𝕤}_{x}$ *respectively, the energy consumption of each node is* $W_{tot}$*, and the energy supplied by the battery during the period is* U*. To ensure the energy consumption is less than or equal to the energy supplied,* i.e., $W_{tot}\leq U$*, the probability of logging shall meet the following conditions:*

(50) $$\beta \geq 1-\frac{U-\nu_{3}\left(\varpi_{R}^{x}-f\right)-\nu_{4}\left(\varpi_{T}^{x}-g\right)-c}{\mu_{3}\left(\varpi_{R}^{x}-f\right)+\mu_{4}\left(\varpi_{T}^{x}-g\right)},when\;\alpha \;\mathrm{is}\;a\;\mathrm{fixed}\;\mathrm{value}$$

*where*

(51) $$\{\begin{array}{c} \varpi_{R}^{x}={𝓅}_{r}T_{d}+\left({𝓅}_{r}T_{p}+{𝓅}_{t}T_{\mathrm{al}}\right)\\ \varpi_{T}^{x}={𝓅}_{t}T_{d}+\frac{\left(1-D_{\mathrm{com}}\right)t_{\mathrm{com}}}{2\left(T_{p}+T_{\mathrm{al}}\right)}\left({𝓅}_{t}T_{p}+{𝓅}_{r}T_{\mathrm{al}}\right)\\ c={𝓅}_{r}D_{\mathrm{com}}+{𝓅}_{t}\left(1-D_{\mathrm{com}}\right)\\ f=\frac{\left[(T_{\mathrm{al}}+T_{d}){𝓅}_{s}+{𝓅}_{r}T_{p}\right]}{t_{\mathrm{com}}}\\ g=\frac{\left\{{𝓅}_{s}\left[\frac{\left(1-D_{\mathrm{com}}\right)t_{\mathrm{com}}}{2}+T_{p}+T_{\mathrm{al}}\right]+{𝓅}_{r}T_{p}\right\}}{t_{\mathrm{com}}}\\ \mu_{3}=\overset{z}{\underset{k=2}{\sum }}\frac{l+kr}{l}b\alpha \times \overset{k}{\underset{i=1}{\sum }}p^{i}\\ \mu_{4}=\overset{z}{\underset{k=1}{\sum }}\frac{l+kr}{l}b\alpha \times \overset{k}{\underset{i=0}{\sum }}p^{i}\\ \nu_{3}=\frac{\left(l+r\right)}{l}\left(m+b\alpha \right)p+\overset{z}{\underset{k=2}{\sum }}\frac{l+kr}{l}mp^{k}\\ \nu_{4}=m+b\alpha +\overset{z}{\underset{k=1}{\sum }}\frac{l+kr}{l}mp^{k} \end{array}$$

**Proof.**  The formula can be obtained according to the energy consumption model: $W_{\mathrm{tot}}=\varpi_{\mathrm{LPL}}^{x}+\varpi_{R}^{x}{𝕣}_{x}+\varpi_{T}^{x}{𝕤}_{x}$. To ensure that the energy consumption is less than the supplied energy, i.e., $W_{\mathrm{tot}}\leq U$, the following formula is obtained:

(52) $${𝓅}_{r}D_{\mathrm{com}}+{𝓅}_{t}\left(1-D_{\mathrm{com}}\right)-\left\{{𝓅}_{s}\left[\frac{\left(1-D_{\mathrm{com}}\right)t_{\mathrm{com}}}{2}+T_{p}+T_{\mathrm{al}}\right]+{𝓅}_{r}T_{p}\right\}\frac{{𝕤}_{x}}{t_{\mathrm{com}}}\;-\left[(T_{\mathrm{al}}+T_{d}){𝓅}_{s}+{𝓅}_{r}T_{p}\right]\frac{{𝕣}_{x}}{t_{\mathrm{com}}}+\varpi_{R}^{x}{𝕣}_{x}+\varpi_{T}^{x}{𝕤}_{x}\leq E$$

Let $c={𝓅}_{r}D_{\mathrm{com}}+{𝓅}_{t}\left(1-D_{\mathrm{com}}\right)$, $f=\frac{\left[(T_{\mathrm{al}}+T_{d}){𝓅}_{s}+{𝓅}_{r}T_{p}\right]}{t_{\mathrm{com}}}$, $g=\frac{\left\{{𝓅}_{s}\left[\frac{\left(1-D_{\mathrm{com}}\right)t_{\mathrm{com}}}{2}+T_{p}+T_{\mathrm{al}}\right]+{𝓅}_{r}T_{p}\right\}}{t_{\mathrm{com}}}$, 

Then:

(53) $$c+\left(\varpi_{R}^{x}-f\right){𝕣}_{x}+\left(\varpi_{T}^{x}-g\right){𝕤}_{x}\leq U$$

According to Theorem 1:

(54) $${𝕣}_{x}=\frac{\left(l+r\right)}{l}\times \left(m+b\alpha \right)p+\overset{z}{\underset{k=2}{\sum }}\frac{\left(l+kr\right)}{l}\times \left[mp^{k}+\left(1-\beta \right)b\alpha \overset{k}{\underset{i=1}{\sum }}p^{i}\right]$$

(55) $${𝕤}_{x}=\left(m+b\alpha \right)+\overset{z}{\underset{k=1}{\sum }}\frac{\left(l+kr\right)}{l}\times \left[mp^{k}+\left(1-\beta \right)b\alpha \overset{k}{\underset{i=0}{\sum }}p^{i}\right]$$

Similarly, treating α as a fixed value, transpose Equation (54):

(56) $${𝕣}_{x}=\left[\overset{z}{\underset{k=2}{\sum }}\frac{l+kr}{l}b\alpha \times \overset{k}{\underset{i=1}{\sum }}p^{i}\right]\times \left(1-\beta \right)+\frac{\left(l+r\right)}{l}\left(m+b\alpha \right)p+\overset{z}{\underset{k=2}{\sum }}\frac{l+kr}{l}mp^{k}$$

Let $\mu_{3}=\Sigma_{k=2}^{z}\frac{l+kr}{l}b\alpha \times \Sigma_{i=1}^{k}p^{i}$, $\nu_{3}=\frac{\left(l+r\right)}{l}\left(m+b\alpha \right)p+\Sigma_{k=2}^{z}\frac{l+kr}{l}mp^{k}$.

Transpose Equation (55):

(57) $${𝕤}_{x}=\left[\overset{z}{\underset{k=1}{\sum }}\frac{l+kr}{l}b\alpha \times \overset{k}{\underset{i=0}{\sum }}p^{i}\right]\times \left(1-\beta \right)+m+b\alpha +\overset{z}{\underset{k=1}{\sum }}\frac{l+kr}{l}mp^{k}$$

Let $\mu_{4}=\Sigma_{k=1}^{z}\frac{l+kr}{l}b\alpha \times \Sigma_{i=0}^{k}p^{i}$, $\nu_{4}=m+b\alpha +\Sigma_{k=1}^{z}\frac{l+kr}{l}mp^{k}$.

Substitute ${𝕣}_{x}=\mu_{3}\left(1-\beta \right)+\nu_{3}$ and ${𝕤}_{x}=\mu_{4}\left(1-\beta \right)+\nu_{4}$ in Equation (53):

(58) $$c+\left(\varpi_{R}^{x}-f\right)\left[\mu_{3}\left(1-\beta \right)+\nu_{3}\right]+\left(\varpi_{T}^{x}-g\right)\left[\mu_{4}\left(1-\beta \right)+\nu_{4}\right]\leq U.$$

Transpose the formula:

(59) $$\beta \geq 1-\frac{U-\nu_{3}\left(\varpi_{R}^{x}-f\right)-\nu_{4}\left(\varpi_{T}^{x}-g\right)-c}{\mu_{3}\left(\varpi_{R}^{x}-f\right)+\mu_{4}\left(\varpi_{T}^{x}-g\right)}$$

**Figure 10:**
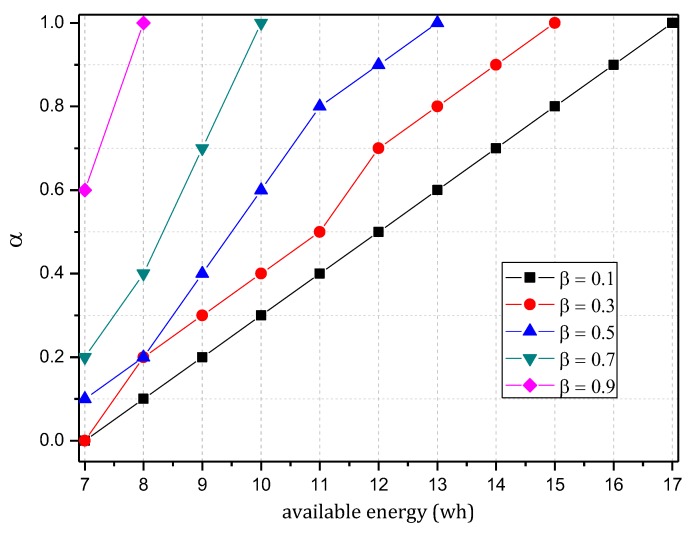
Values of probability of marking the node 1 hop from the sink when the probability of logging is a fixed value (β=0.1, 0.3, 0.5, 0.7, 0.9) and the available energy is (7, 8,⋯, 17).

**Figure 11:**
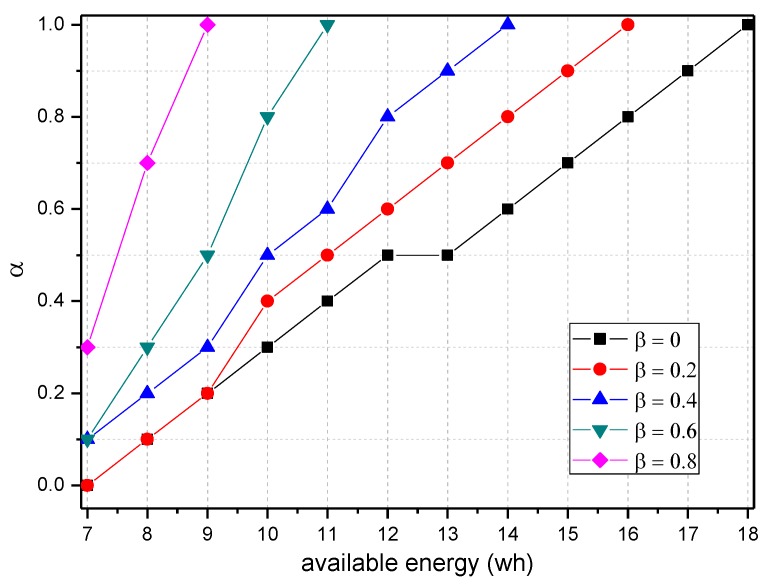
Values of probability of marking the node 1 hop from the sink when the probability of logging is a fixed value (β=0, 0.2, 0.4, 0.6, 0.8) and the available energy is (7, 8,⋯, 17).

**Figure 12:**
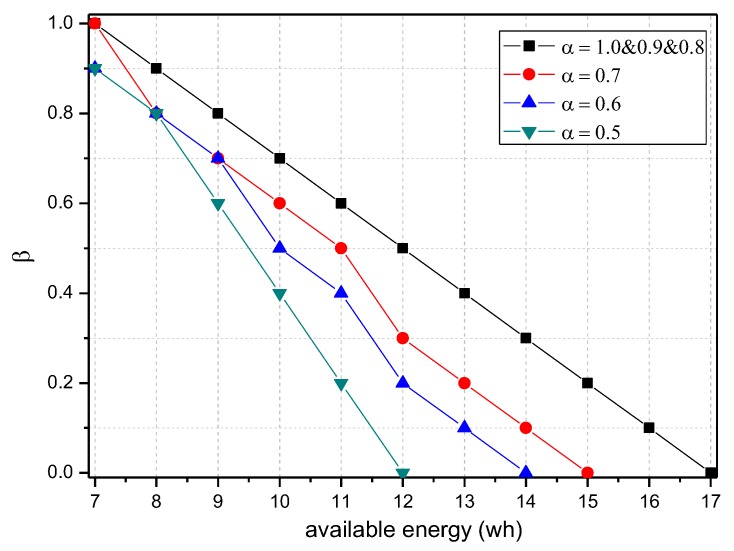
Values of probability of logging the node 1 hop from the sink when the probability of marking is a fixed value (α=1.0, 0.9,0.8,0.7,0.6,0.5) and the available energy is (7, 8,⋯, 17).

**Figure 13:**
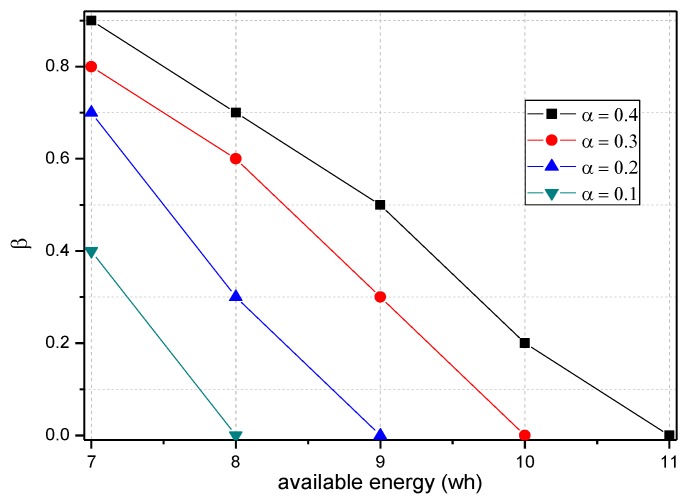
Values of probability of logging the node 1 hop from the sink when the probability of marking is a fixed value (α=0.1, 0.2, 0.3, 0.4) and the available energy is (7, 8,⋯, 17).

Combining Theorem 2, to maximize the utilization of the level of battery remaining and solar radiation, we should improve network security as much as possible but control the energy consumption within the available energy, i.e., use a larger α and smaller β as possible. According to Theorems 4 and 5, we can make Figure 10, Figure 11, Figure 12 and Figure 13. Figure 10 and Figure 11 shows the maximum value of α under different fixed β when the available energy is 7~17 wh. Figure 12 and Figure 13 shows the minimum value of β under different fixed α when the available energy is 7~17 wh.

According to Figure 10, Figure 11, Figure 12 and Figure 13, when available energy = 7, there is one sequence containing multiple pairs (α, β) meeting the requirement of Theorem 2 as shown in the following table. Similarly, when available energy = 8, …, a corresponding sequence can be found. Our purpose is to find out the pair that enables the sink to receive the largest amount of notification, so this paper takes available energy = 7, 8, 9, 10, and 11 as examples, removes some pairs that are obviously not the optimal options and reserves some pairs that are possibly optimal.

In this paper, convergence time is an important index to evaluate the TBSR scheme. Convergence time refers to the time taken by the whole synchronization process of routing information after the routers find the change in the topology structure of the network. Actually, in the TBSR scheme, convergence time is largely determined by the amount of notification that the sink can collect. When victims are attacked, they will consult the information of the upstream nodes and reconstruct the attack path in the traceback request in the form of broadcasting the malicious packet information. If the sink receives more notification, the victims can collect sufficient notification to determine the malicious node in a shorter time. In contrast, the victims must wait for another attack of the malicious node. Moreover, the data packet attacked must be marked, and the notification must be transmitted to the victims. Clearly, the more notification the sink of the network receives, the better the convergence time index will be. Therefore, this paper uses the amount of notification received by the sink to reflect the convergence time. The following Theorem 5 calculates the amount of notification received by the sink.

**Theorem** **5.** *For a planar network, assuming the radius of the whole network is* R*, the transmission radius of a data packet is* r*, the success rate of each hop is* p*, the length of the data packet is* m *bits, the length of digital marking is* b *bits, and the node sending the data packet is logged with the probability of* β *after one hop. The amount of notification received by the sink is as follows*:

(60) $$M_{\left(\alpha ,\;\beta \right)}=\rho \pi r^{2}\left\{b\alpha p\left[1+\left(1-\beta \right)\overset{z}{\underset{k=1}{\sum }}\left(1+k\right)\right]+\overset{z}{\underset{k=1}{\sum }}b\alpha p^{k+1}\left(1-\beta \right)\overset{z}{\underset{i=k}{\sum }}\left(1+i\right)\right\}\;|z=\lfloor \frac{R-l}{r}\rfloor$$

**Proof.**  The amount of notification received by the sink is the product of the amount of data sent by the node 1 hop from the sink and p. The amount of notification sent by the node that is l from the sink is as follows:

(61) $${𝕤}_{mark}=b\alpha +\left(l+r\right)\left(1-\beta \right)\left(b\alpha p+b\alpha \right)+\left(l+2r\right)\left(1-\beta \right)\left(b\alpha p^{2}+b\alpha p+b\alpha \right)+\cdots +\;\left(l+zr\right)\left(1-\beta \right)\left(b\alpha p^{z}+\cdots +b\alpha p^{2}+b\alpha p+b\alpha \right)=b\alpha \left[1+\left(1-\beta \right)\Sigma_{k=1}^{z}\frac{l+kr}{l}\right]+\;\Sigma_{k=1}^{z}b\alpha p^{k}\left(1-\beta \right)\Sigma_{i=k}^{z}\frac{l+ir}{l}|\;z=\lfloor \frac{R-l}{r}\rfloor$$

l=hr+x, so the above formula can be converted to the following:

(62) 𝕤markh= bα[1+(1−β)∑k=1z(1+kh)]+∑k=1zbαpk(1−β)∑i=kz(1+ih) |z=⌊R−lr⌋

Therefore, the amount of notification sent by each node of *h* = 1 is as follows:

(63) $${𝕤}_{mark_{1}}=\;b\alpha \left[1+\left(1-\beta \right)\overset{z}{\underset{k=1}{\sum }}\left(1+k\right)\right]+\overset{z}{\underset{k=1}{\sum }}b\alpha p^{k}\left(1-\beta \right)\overset{z}{\underset{i=k}{\sum }}\left(1+i\right)\;|z=\lfloor \frac{R-l}{r}\rfloor$$

The amount of notification received by the sink in the whole area is as follows:

(64) $$M=\rho \pi r^{2}\left\{b\alpha p\left[1+\left(1-\beta \right)\overset{z}{\underset{k=1}{\sum }}\left(1+k\right)\right]+\overset{z}{\underset{k=1}{\sum }}b\alpha p^{k+1}\left(1-\beta \right)\overset{z}{\underset{i=k}{\sum }}\left(1+i\right)\right\}\;|z=\lfloor \frac{R-l}{r}\rfloor$$

The above analysis of the amount of notification received by the sink is actually an analysis of convergence time because the more notification the sink of the network receives, the shorter convergence time will be. In the TBSR scheme, the storage space of the node will also be considered in addition to convergence time. If too much notification is stored in a node, the strategy is not perfect. The following calculates the amount of notification stored in each node.

**Theorem** **6.** *For a planar network, assuming the length of data packet is* m *bits and the length of digital marking is* b *bits, the node sending the data packet and notification will be logged with the probability of* β *after one hop, and the amount of notification stored in each node is as follows*:

(65) $$\xi =\int_{0}^{t}\beta b\alpha pdt$$

**Proof.**  It can be obtained from the proof process of Theorem 1 that each node only logs the notification of the starting node of the last hop. The amount of notification sent by the starting node of last hop and received by the current node is bαp; the current node is logged with the probability of β, so the amount of notification stored in a node at a certain moment is βbαp. The accumulated amount of notification at any time in a day should be calculated through time integration, i.e., $\xi =\int_{0}^{t}\beta b\alpha pdt$.

As shown in the theorem, in the TBSR scheme, the logged notification is distributed over the whole network, so that only a small number of notifications are stored in each node and the stored notification will be sent out the next day, so the node has a light load, which proves the good performance of the TBSR scheme.

According to Theorem 5, we can select one from all satisfactory (α, β) pairs to achieve the best network security, i.e., the (α, β) pair with the maximum convergence time. As shown in Table 2 in the last section, five (α, β) pairs meet the requirement of available energy = 7 wh. After calculating the convergence time of the five pairs, we determine that (0.6, 0.9) has the maximum convergence time and best security. Similarly, we can also find the (α, β) that achieves the best security when the available energy is another value. The following Table 3 shows the (α, β) with best security obtained through calculation.

The above is our analysis on two indexes of the TBSR scheme—convergence time and storage space of a node. In the following part, we will analyze the performance of the TBSR scheme based on the actual situation.

## 5. Performance Analysis

### 5.1. Experimental Result

The following Table 4 shows the experiment conditions and selected parameters:

We assume the length of the data packet after aggregation as 500 and the length of notification as 100. We select the solar energy receiver in dimensions of 10 cm × 20 cm. The initial level and maximum level of the node battery are 55 wh and 111 wh, respectively. 

**Figure 14:**
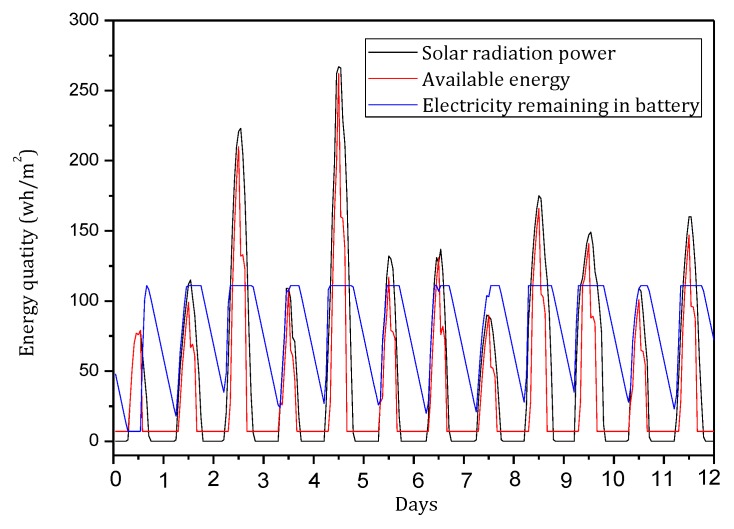
Solar radiation energy, available energy and remaining energy of battery for the 12 days.

First, we select the first day from the data of the Solar Radiation Laboratory of Texas, USA [60], based on the TBSR scheme (The TBSR scheme requires the first day to be the day with the least solar radiation in recent years), select the remaining 11 days randomly and draw Figure 14 under the above conditions. As shown in the figure, the minimum level of the node battery remaining is 7 wh according to the energy consumption plan of the TBSR scheme, and it appears on the first day. On any later day, the remaining energy of the node battery is over 20 wh, and the battery can be fully charged every day.

**Figure 15:**
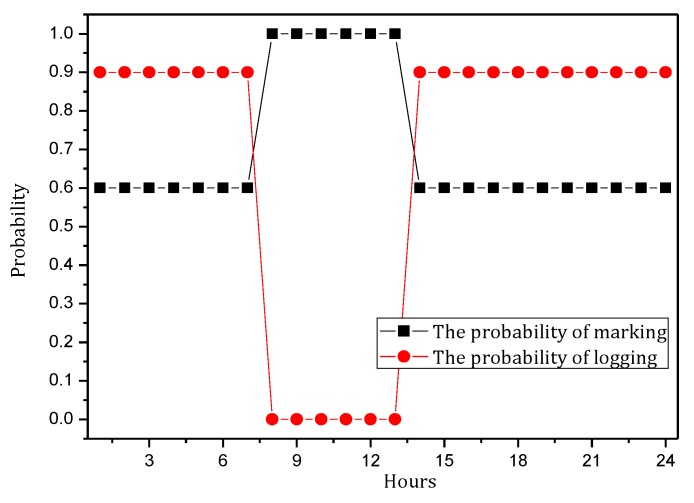
Change of probability of marking and logging of the node 1 hop from the sink in the first day.

**Figure 16:**
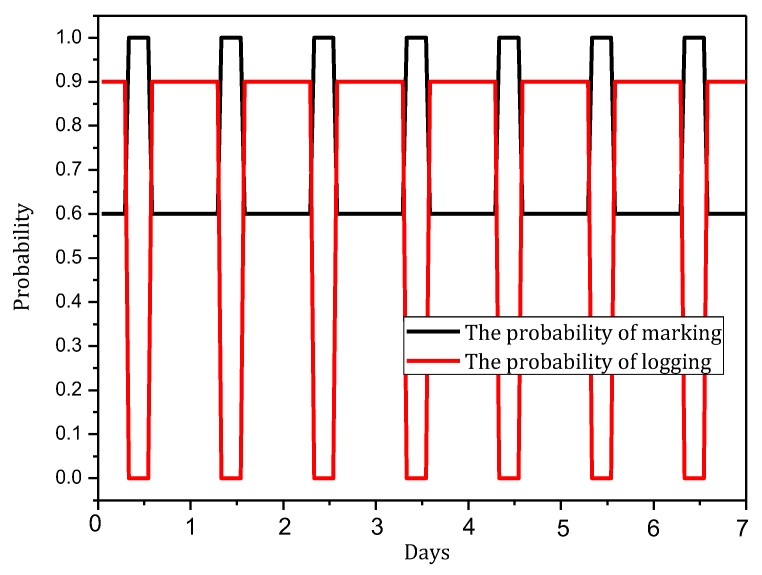
Change of probability of marking and logging of the node 1 hop from the sink in one week.

Figure 15 and Figure 16 show the analysis conducted based on Figure 14. The nodes closer to the sink load the greatest amounts of data and consume the most energy, so we analyze the node 1 hop from the sink first. Under the experiment conditions of Figure 14 and according to the TBSR scheme, we can obtain the probability of marking and logging of the node 1 hop from the sink at different times.

Figure 15 shows the change of probability of marking and logging of the node 1 hop from the sink in each hour of the first day. According to Figure 15, from 1 to 7 o’clock, (α, β)=(0.6, 0.9), from 8 to 13 o’clock, (α, β)=(1, 0) and from 14 to 24 o’clock, (α, β)=(0.6, 0.9). Therefore, (0.6, 0.9) is the best solution to ensure network security when the energy consumption is 7 wh and (1, 0) the best solution when the remaining battery level is high.

Figure 16 shows the change of probability of marking and logging of the node 1 hop from the sink in one week. The value of (α, β) is similar to that of the first day.

**Figure 17:**
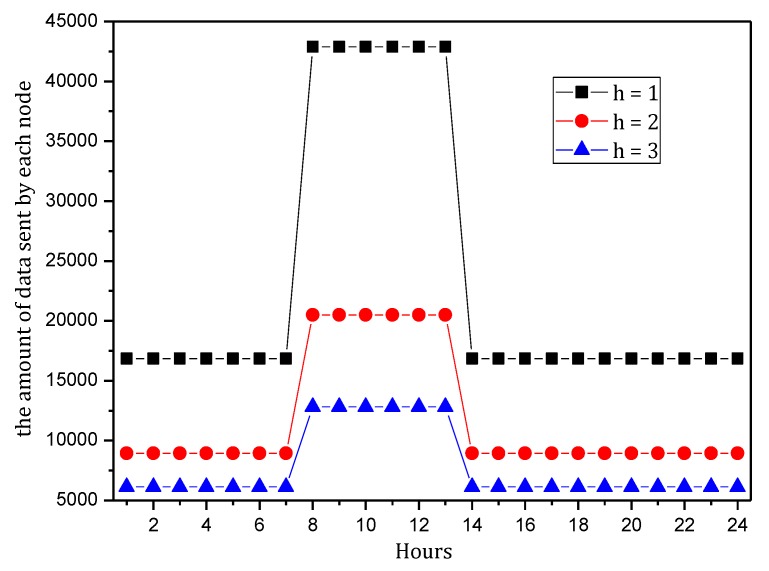
Amount of data sent by each node 1, 2 and 3 hops from the sink in the first day.

**Figure 18:**
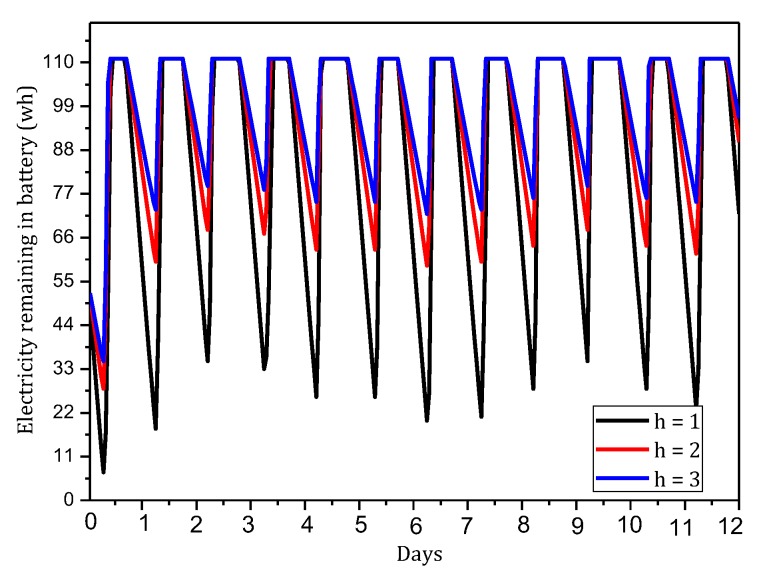
Change of remaining battery level of each node 1, 2 and 3 hops from the sink in 12 days.

**Figure 19:**
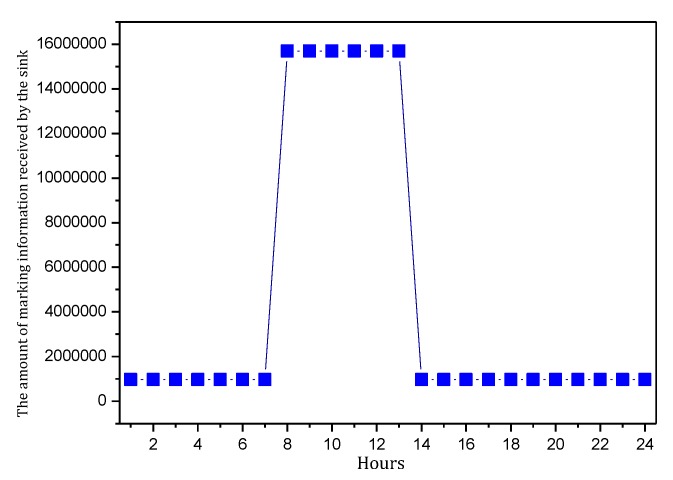
Change of the amount of notification received by the sink on the first day.

Based on Figure 14, Figure 15 and Figure 16 and Theorem 1, we can draw the amount of data sent by each node 1, 2 and 3 hops from the sink in the first day as shown in Figure 17. Figure 17 shows that the closer the node is to the sink; the more data is sent. According to Figure 15, in the period of 8–13 h, the probability of marking is high and the probability of logging is low. Therefore, in Figure 17, the amount of data during this period of 8–13 h is significantly greater than at other times. In order to maintain the level of battery remaining above 0 or a lower limit at any time, we must consider the change of the remaining battery level of the nodes. As shown in Figure 18, in 12 days, taking the nodes 1, 2 and 3 hops from the sink as examples, the level of battery remaining is always over 0 wh and the battery can be charged once a day. Based on the observation of Figure 18 and a further analysis, we find that the nodes farther from the sink have more remaining battery level at any time because they load fewer amounts of data and consume less energy. Therefore, if the battery of the node 1 hop from the sink can be kept in use, the same parameters can be applied to other nodes of the network to avoid the death of these nodes. According to Figure 17, we can also draw the change of the amount of notification received by the sink in the first day as shown in Figure 19. From 8 to 13 o’clock, the sink clearly receives a large amount of notification, and the network achieves favorable security.

### 5.2. Performance Comparison with the Traceback with Stationary Parameter Scheme

In the TBSR scheme, the probability of marking and logging will vary with the available energy, which results in effectively utilizing the energy and thereby improving network security and reliability. The traceback scheme in the paper adopts the fixed probability of marking and logging (traceback with stationary parameter scheme, known as the TWSP scheme). Next, we compare the performance of these two schemes in four aspects.

(1) Comparison of convergence time

**Figure 20:**
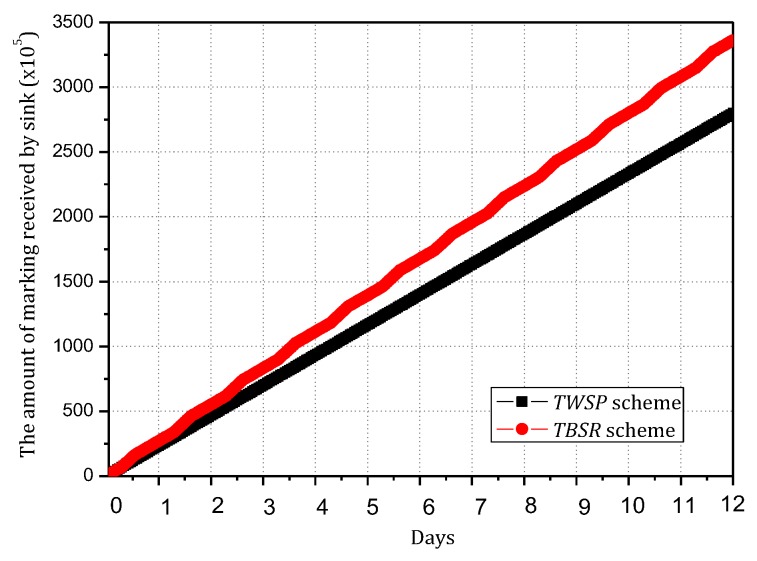
Comparison of the accumulated amount of notification received by the sink in the Trust-Based Secure Routing (TBSR) scheme and the Traceback with Stationary Parameter (TWSP) scheme.

**Figure 21:**
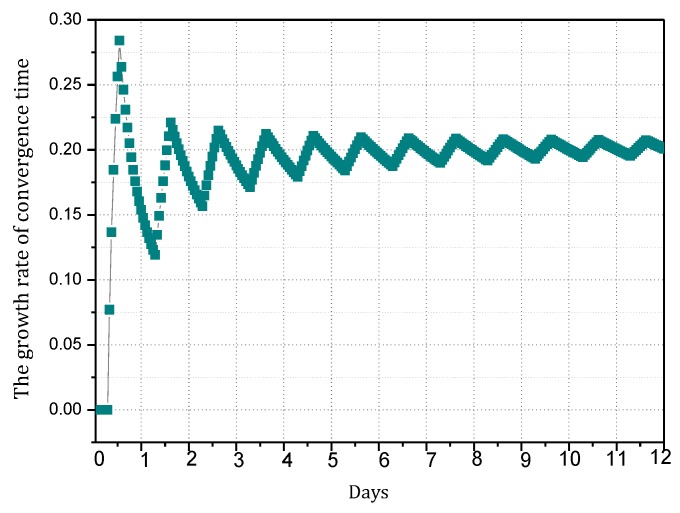
Time-based Change of accumulated amount of notification received by the sink in the TBSR scheme compared with that in the TWSP scheme.

**Figure 22:**
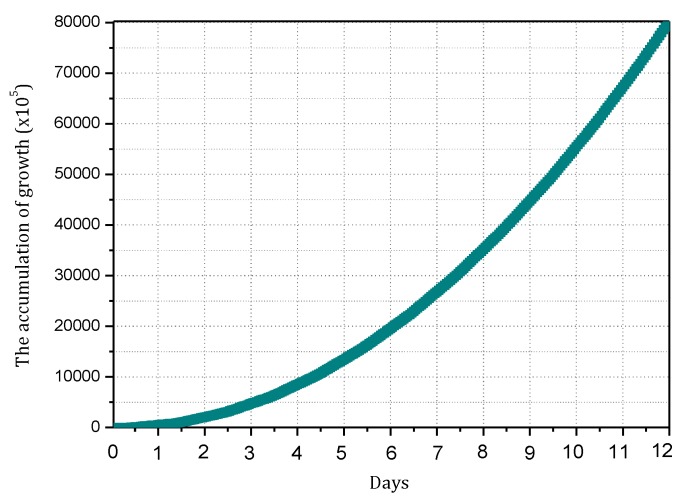
Shows the increase of the accumulated amount of notification received by the sink as time elapses in the TBSR scheme compared with that in the TWSP scheme.

The following compares the amount of marking received cumulatively by sink (actually also the comparison of convergence time):

Figure 20, Figure 21 and Figure 22 shows the comparison of convergence time between the TBSR scheme and the TWSP scheme. As shown in Figure 20, at the same moment, the sink receives more notification in the TBSR scheme than in the TWSP scheme, and as time elapses, the accumulated amount of notification received by the sink under the two schemes presents an increasing difference. Figure 21 shows the amount of notification received by the sink in the TBSR scheme further calculated based on Figure 20, which increases approximately 20% compared with the amount of notification received by the sink in the TWSP scheme. Figure 22 shows the length of accumulated notification received by the sink in the TBSR scheme after 12 days that is further calculated based on Figure 20, which is $8\times 10^{9}$ greater than that in the TWSP scheme. When the sink in the network receives more notification, the convergence time index will be more favorable and the network will be more reliable, so the TBSR scheme has higher security.

(2) Comparison of accumulated energy consumption of nodes

**Figure 23:**
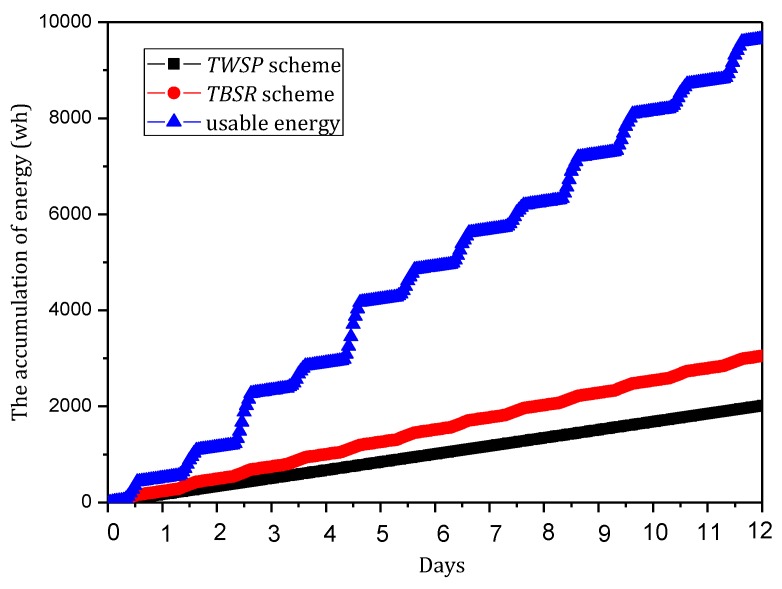
Comparison of the accumulated energy consumption and available energy in the TBSR scheme and the TWSP scheme.

**Figure 24:**
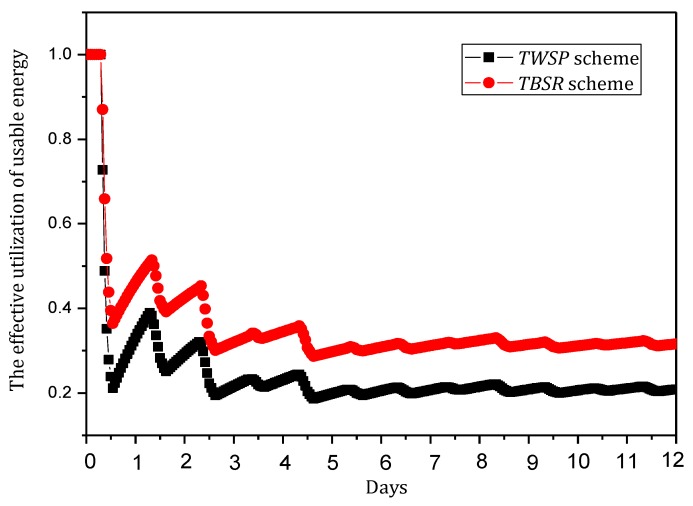
Comparison of energy availability in the TBSR scheme and the TWSP scheme.

**Figure 25:**
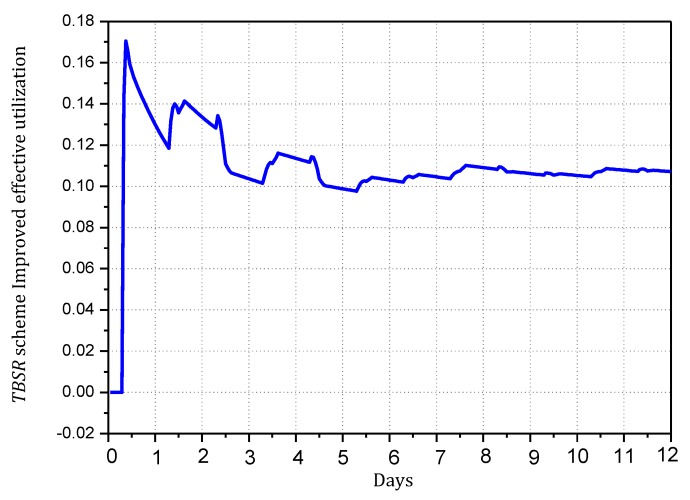
Increase of energy availability in the TBSR scheme compared with that of the TWSP scheme.

Figure 23, Figure 24 and Figure 25 compare the accumulated energy consumption in the TBSR scheme and the TWSP scheme.

As shown in Figure 23, at the same moment, the accumulated energy consumption in the TBSR scheme as time elapses is greater than that in the TWSP scheme because in the TBSR scheme, the probabilities of marking and logging are changeable. During the several hours in a day when the available energy is sufficient, we improve the probability of marking and reduce the probability of logging, so the node consumes more energy than it consumes at other times. However, in the TWSP scheme, the probabilities of marking and logging are fixed and selected based on the minimum available energy to avoid the death of the node. Further analysis shows that the nodes can make better use of the available energy in the TBSR scheme. As shown in Figure 24, the energy availability of the TWSP scheme is approximately 20%, but the energy availability of the TBSR scheme is as high as greater than 30%. Figure 25 shows the increase of energy availability in the TBSR scheme compared with that in the TWSP scheme. Figure 25 clearly shows that the availability is increased by approximately 11%. Therefore, the TBSR scheme has higher energy availability and better performance.

(3) Comparison of amount of notification stored in nodes

**Figure 26:**
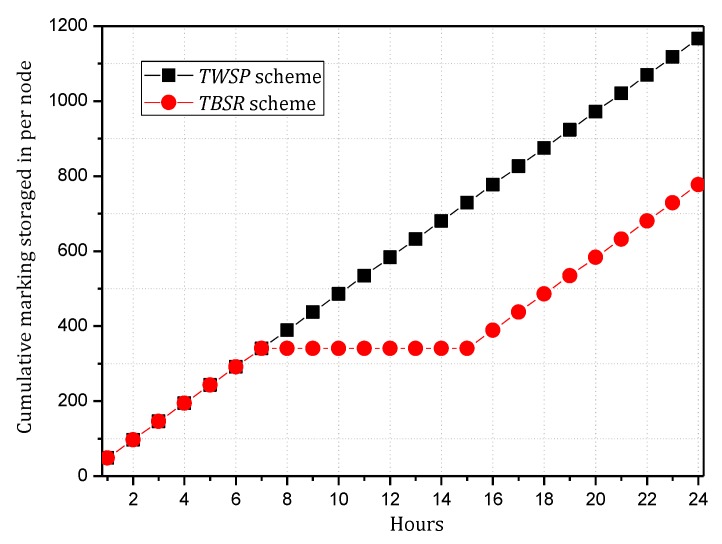
Comparison of the amount of notification stored in nodes in the TBSR scheme and the TWSP scheme.

**Figure 27:**
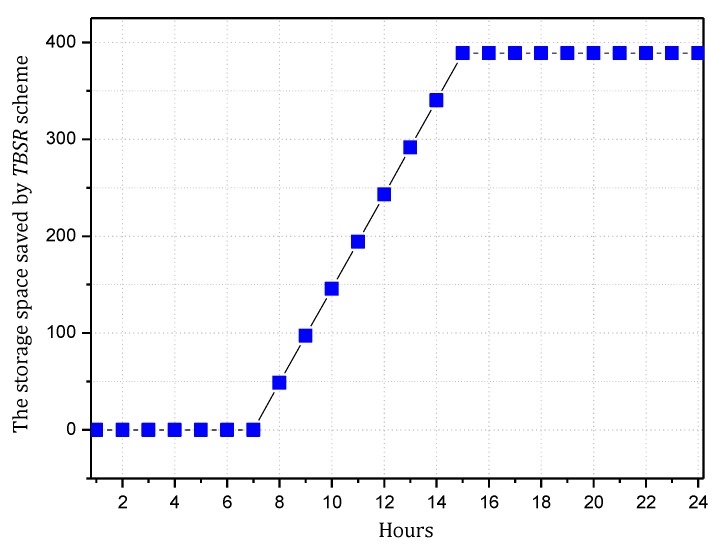
Storage space saved by the TBSR scheme in one day.

Figure 26 and Figure 27 compare the amount of notification stored in nodes in the TBSR scheme and the TWSP scheme.

As shown in Figure 26, in the TBSR scheme, the amount of stored notification does not increase during a certain period because in this period, the probability of logging is 0 and no notification is stored. Figure 27 shows the storage space saved for nodes in the TBSR scheme compared with the TWSP scheme. As the figure shows, the length of notification saved by the TBSR scheme in one day is near 400. Therefore, the TBSR scheme can save more storage space and provide better performance.

(4) Comparison of success rate of routing

This section analyzes the calculation of arrival rates in the TWSP scheme and the TBSR scheme and a performance comparison of two schemes. As mentioned above, more notification will result in shorter convergence time and higher security during the traceback. In this case, the sink can find the secure transmission path more easily using the received notification. In other words, a greater probability of marking leads to higher trust, i.e., a greater success rate of the transmission of each hop.

**Theorem** **7.** *Assume the source node sending the data packet has a distance of* h *hops from the sink and the number of hops of the data packet during horizontal routing is a random number in* {1,𝒹}*. The expected number of hops of the horizontal routing is* 𝒹/2*; therefore, the average number of hops for sending the data packet to the sink is* h+𝒹/2*. The routing process of a notice message is highly similar to that of a data packet, so the average number of hops for sending the notice message to the sink is also* h+𝒹/2*. Assuming the number of sent data packets is* ℳ*, the number of notice messages must be the same as that of data packets,* i.e., ℳ*. We adopt the TWSP scheme and the TBSR scheme for routing, respectively. Assuming the success rate of each hop in the TWSP scheme is* p *and the trust increased by the successful transmission of each hop in the TBSR scheme is* ∂*, the arrival rate of a data packet is* $\pi_{h}^{1}$ *and* $\pi_{h}^{2}$ *respectively in the TWSP scheme and the TBSR scheme*:

(66) $$\{\begin{array}{c} \pi_{h}^{1}=1-{\left(1-p^{h+𝒹/2}\right)}^{2ℳ}\\ \pi_{h}^{2}=1-{\left[1-{\left(p+\partial \right)}^{h+𝒹/2}\right]}^{2ℳ} \end{array}$$

**Proof.**  In the TWSP scheme, the node has a distance of h+𝒹/2 hops from the sink, and the success rate of transmission of each hop is p. Therefore, the probability of each data packet or notification of successfully reaching the sink is $p^{h+𝒹/2},$ the probability of each data packet or notification failing to reach the sink is $1-p^{h+𝒹/2}$, the probability that all ℳ data packets and ℳ notification fail to reach the sink is ${\left(1-p^{h+𝒹/2}\right)}^{2ℳ}$, and the probability that the sink receives at least one data packet or notification is $1-{\left(1-p^{h+𝒹/2}\right)}^{2ℳ}$. Therefore, the success rate of routing is $\pi_{h}^{1}=1-{\left(1-p^{h+𝒹/2}\right)}^{2ℳ}$. In the TBSR scheme, the success rate of transmission of each hop is p+∂. Similarly, the success rate of routing can be determined: $\pi_{h}^{2}=1-{\left[1-{\left(p+\partial \right)}^{h+𝒹/2}\right]}^{2ℳ}$.

**Figure 28:**
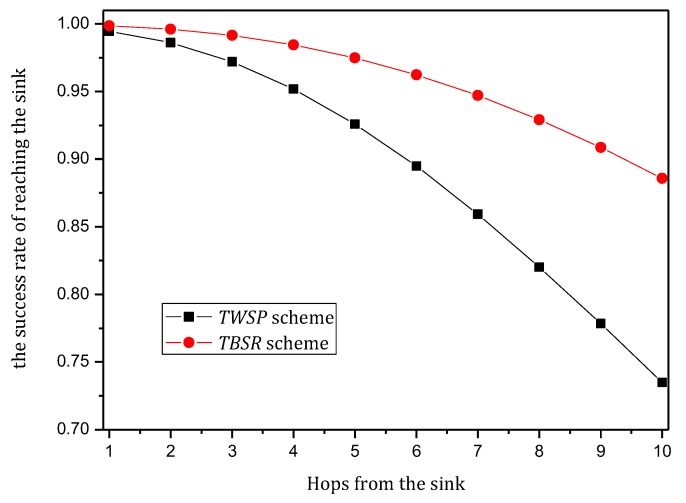
Comparison of success rate of routing of the TWSP scheme and the TBSR scheme when p=0.90, ∂=0.03 and ℳ=2,𝒹=4.

**Figure 29:**
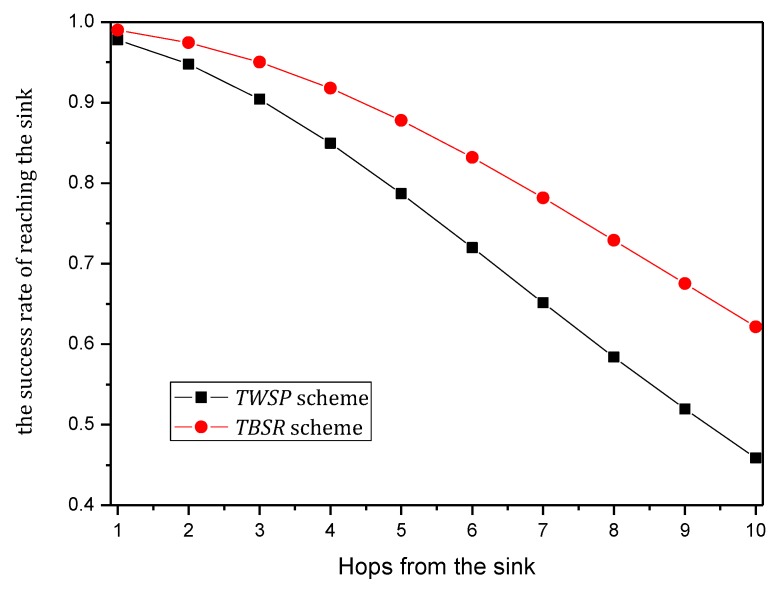
Comparison of success rate of routing of the TWSP scheme and the TBSR scheme when p=0.85, ∂=0.03 and ℳ=2,𝒹=4.

Figure 28 and Figure 29 compare the success rate of routing in the TBSR scheme and the TWSP scheme. As the figure shows, the difference between the success rates of routing in the two schemes will be greater for nodes farther from the sink. As shown in Figure 28, when the nodes 10 hops from the sink send packets, the success rate of routing in the TBSR scheme is 15.09% higher than that in the TWSP scheme. Similarly, as shown in Figure 29, when nodes 10 hops from the sink send data packets, the success rate of routing in the TBSR scheme is 16.30% greater than that in the TWSP scheme.

According to the results of comparison of the above four aspects, the amount of notification received by the sink increases by approximately 20% in the TBSR scheme compared with that in the TWSP scheme, the energy availability increases by approximately 11%, the maximum storage capacity required by the node decreases by 33.33%, and the success rate of routing increases by approximately 16.30%. Therefore, the TBSR scheme has better performance.

## 6. Conclusions

In this paper, a secure routing scheme using the traceback approach for energy-harvesting sensor networks is proposed to maximize the use of available energy to improve data security and integrity. First, the aggregate signature approach is used to aggregate data and maintain data integrity. Then, a data and notification disjoint routing approach is proposed to improve the probability of the data reaching the sink safely. However, a scheme based only on these two approaches cannot determine the location of a malicious node. Therefore, in this paper, we propose a scheme integrating the traceback scheme and combining the ID-based aggregate signature approach with multi-path routing of data packets and notification, which not only reduces the energy consumption but also ensures data security and integrity. The improvements of the past traceback scheme proposed by this paper in the TBSR include the following: when available energy of nodes is sufficient, a higher probability of marking and a lower probability of logging are used. Thus, the sink can obtain more notification, which will improve network security. Because if the probability of marking is higher, the number of marked nodes on the data packet routing path will be more, and the sink will be more likely to trace back the data packet routing path and find malicious nodes according to this notification. When data packets are routed again, they tend to bypass these malicious nodes, which make the success rate of routing higher and lead to improved network security. In contrast, when available energy of nodes is insufficient, a lower probability of marking and a higher probability of logging are used, which stores the notification on the nodes of the network instead of sending it to the sink immediately. Thus, when the level of battery remaining is low, less data is transmitted in the network, which saves energy. When the level of battery remaining is enough, the notification logged on the nodes of the network will be transmitted to the sink. This approach significantly improves the overall security of the system and energy availability. Finally, the TBSR scheme uses the malicious node location function based on traceback to reduce the trust of the malicious node and to guide the data to avoid the nodes with low trust to further improve the system security. The results of our strict theoretic analysis show that, compared with the ordinary traceback scheme (TWSP scheme), the TBSR scheme can increase the amount of notification received by the sink by approximately 20%, increase the energy availability by approximately 11%, reduce the maximum storage capacity of the node by 33.3% and improve the routing success rate by approximately 16.30%. It therefore has better performance.

## Figures and Tables

**Table 1:** Network parameters.

Symbol	Description	Value
$t_{com}$	Duration of communication	100 ms
$T_{p}$	Duration of masthead	0.26 ms
$T_{al}$	Duration of confirmation window	0.26 ms
$T_{d}$	Duration of data packet	0.93 ms
${𝒫}_{t}$	Transmission power consumption	0.0511 w
${𝒫}_{r}$	Receiving power consumption	0.0588 w
${𝒫}_{s}$	Sleeping power consumption	2.4 × 10^−7^ w
$\varpi_{LPL}^{x}$	Power required to execute LPL operation (duration of $t_{com}$)	Related to calculation
$\varpi_{R}$	Power of nodes for receiving data packet	Related to calculation
$\varpi_{T}$	Power of nodes for transmitting data packet	Related to calculation
$D_{com}$	Duty cycle	0.5

**Table 2:** Candidate pairs for the node of h=1.

Available Energy (wh)	(α, β)			
7	(0.2,0.7)	(0.3,0.8)	(0.6,0.9)	(1,1)
8	(0.3,0.6)	(0.4,0.7)	(0.7,0.8)	(1,0.9)
9	(0.3,0.3)	(0.5,0.6)	(0.7,0.7)	(1,0.8)
10	(0.4,0.2)	(0.6,0.5)	(0.7,0.6)	(1,0.7)
11	(0.5,0.2)	(0.6,0.4)	(0.7,0.5)	(1,0.6)

**Table 3:** (α, β) with the best security performance when R=200, r=20, ρ=0.5 and the node with h = 1 has different values of available energy.

Available Energy (wh)	α	β	When Convergence Time is min, Sink’s Notification	Storage Space
7	0.6	0.9	971,989.79	48.6
8	0.7	0.8	2,228,412.17	50.4
9	0.7	0.7	3,322,836.25	44.1
10	0.4	0.2	5,025,689.53	7.2
11	1	0.6	6,276,459.91	54.0

**Table 4:** Experiment parameters.

Symbol	Description	Value
m	Length of data packet	500
b	Length of marking	100
p	Success rate of transmission of each hop	0.9
R	Network radius	200 m
r	Emission radius of node	20 m
ρ	Distribution density of node	0.5
$E_{initial}$	the initial level of battery	55 wh
$E_{max}$	the maximum level of battery	111 wh
